# *In silico *identification of a core regulatory network of OCT4 in human embryonic stem cells using an integrated approach

**DOI:** 10.1186/1471-2164-10-314

**Published:** 2009-07-15

**Authors:** Lukas Chavez, Abha S Bais, Martin Vingron, Hans Lehrach, James Adjaye, Ralf Herwig

**Affiliations:** 1 Department of Vertebrate Genomics, Max-Planck-Institute for Molecular Genetics, Ihnestrasse 73, D-14195 Berlin, Germany; 2 Department of Computational Molecular Biology, Max-Planck-Institute for Molecular Genetics, Ihnestrasse 73, D-14195 Berlin, Germany

## Abstract

**Background:**

The transcription factor OCT4 is highly expressed in pluripotent embryonic stem cells which are derived from the inner cell mass of mammalian blastocysts. Pluripotency and self renewal are controlled by a transcription regulatory network governed by the transcription factors OCT4, SOX2 and NANOG. Recent studies on reprogramming somatic cells to induced pluripotent stem cells highlight OCT4 as a key regulator of pluripotency.

**Results:**

We have carried out an integrated analysis of high-throughput data (ChIP-on-chip and RNAi experiments along with promoter sequence analysis of putative target genes) and identified a core OCT4 regulatory network in human embryonic stem cells consisting of 33 target genes. Enrichment analysis with these target genes revealed that this integrative analysis increases the functional information content by factors of 1.3 – 4.7 compared to the individual studies. In order to identify potential regulatory co-factors of OCT4, we performed a *de novo *motif analysis. In addition to known validated OCT4 motifs we obtained binding sites similar to motifs recognized by further regulators of pluripotency and development; e.g. the heterodimer of the transcription factors C-MYC and MAX, a prerequisite for C-MYC transcriptional activity that leads to cell growth and proliferation.

**Conclusion:**

Our analysis shows how heterogeneous functional information can be integrated in order to reconstruct gene regulatory networks. As a test case we identified a core OCT4-regulated network that is important for the analysis of stem cell characteristics and cellular differentiation. Functional information is largely enriched using different experimental results. The *de novo *motif discovery identified well-known regulators closely connected to the OCT4 network as well as potential new regulators of pluripotency and differentiation. These results provide the basis for further targeted functional studies.

## Background

Several studies on reprogramming human somatic cells to induced pluripotent stem cells (iPS) have demonstrated that the transduction of only a few transcription factors (TFs) is sufficient for resetting differentiated cells into a molecular state similar to embryonic stem cells (ESCs). While Takahashi et al. [1] and Wernig et al. [2] obtained iPS cells by transduction of the TFs OCT4, SOX2, KLF4, and C-MYC, Yu et al. [3] achieved similar results with a transcription factor set composed of OCT4, SOX2, NANOG, and LIN28. Only the TFs OCT4 and SOX2 are common in both approaches and Huangfu et al. [4] demonstrated that iPS cells can be derived at higher efficiencies by the transduction of these two factors in combination with the supplementation with the histone deacetylase inhibitor -valproic acid.

The TF OCT4 is known as a key regulator for maintaining pluripotency in the mammalian embryo [5-7]. The HMG-box containing TF SOX2 interacts with OCT4 and the SOX2/OCT4 heterodimer complex is able to promote selective gene activation or repression during mammalian embryogenesis [8-11].

Functional data on OCT4 regulatory action is available from heterogeneous sources: to reveal DNA-Protein binding events of OCT4, SOX2 and of the pluripotency associated TF NANOG, chromatin immuno-precipitation followed by microarray experiments (ChIP-on-chip) has been performed using hESCs [12]. Additionally, sequence motifs have been identified, for example the octamer motif *ATTTGCAT *interacting with POU domain factors like the homeodomain containing TF OCT4 and a motif recognized by the SOX2/OCT4 heterodimer complex [13-15]. Mapping of these known transcription factor binding motifs to the promoter sequences of putative OCT4 target genes provides additional evidence for direct binding events.

Although ChIP-on-chip experiments and sequence-based methods have the ability to detect such putative protein-DNA binding sites, these techniques do not allow inference of directional transcriptional dependencies between DNA binding and the effect on regulation of gene expression. In order to test the regulatory influence of OCT4 to the transcription rate of its target genes, Babaie et al. [16] performed RNA interference-mediated suppression of OCT4 function in the H1 hESC line and analyzed the resulting global gene expression changes by microarray experiments. Transcriptional changes induced by OCT4 knockdown are expected to include genes linked with pluripotency, and genes activated upon differentiation along the trophoblast lineage [16].

ChIP-on-chip experiments, promoter sequence analysis and RNA interference provide complementary pieces of information on transcriptional dependencies. In this study, we performed an integrated analysis of these methods in the context of OCT4 dependent regulation of pluripotency and differentiation along the trophoblast lineage in hESCs in order to construct a core network composed of the genes that were detected by all individual experimental approaches. Using this conservative selection, we observed a 1.3–4.7 fold increase of functional information content compared to single experiment analysis. In order to extend the analysis of OCT4 regulation, we performed a comprehensive *in silico *promoter sequence analysis with the OCT4 target genes and identified binding sites related to potential co-factors of OCT4.

## Results

### Analysis of individual experimental methods

**Figure 1:**
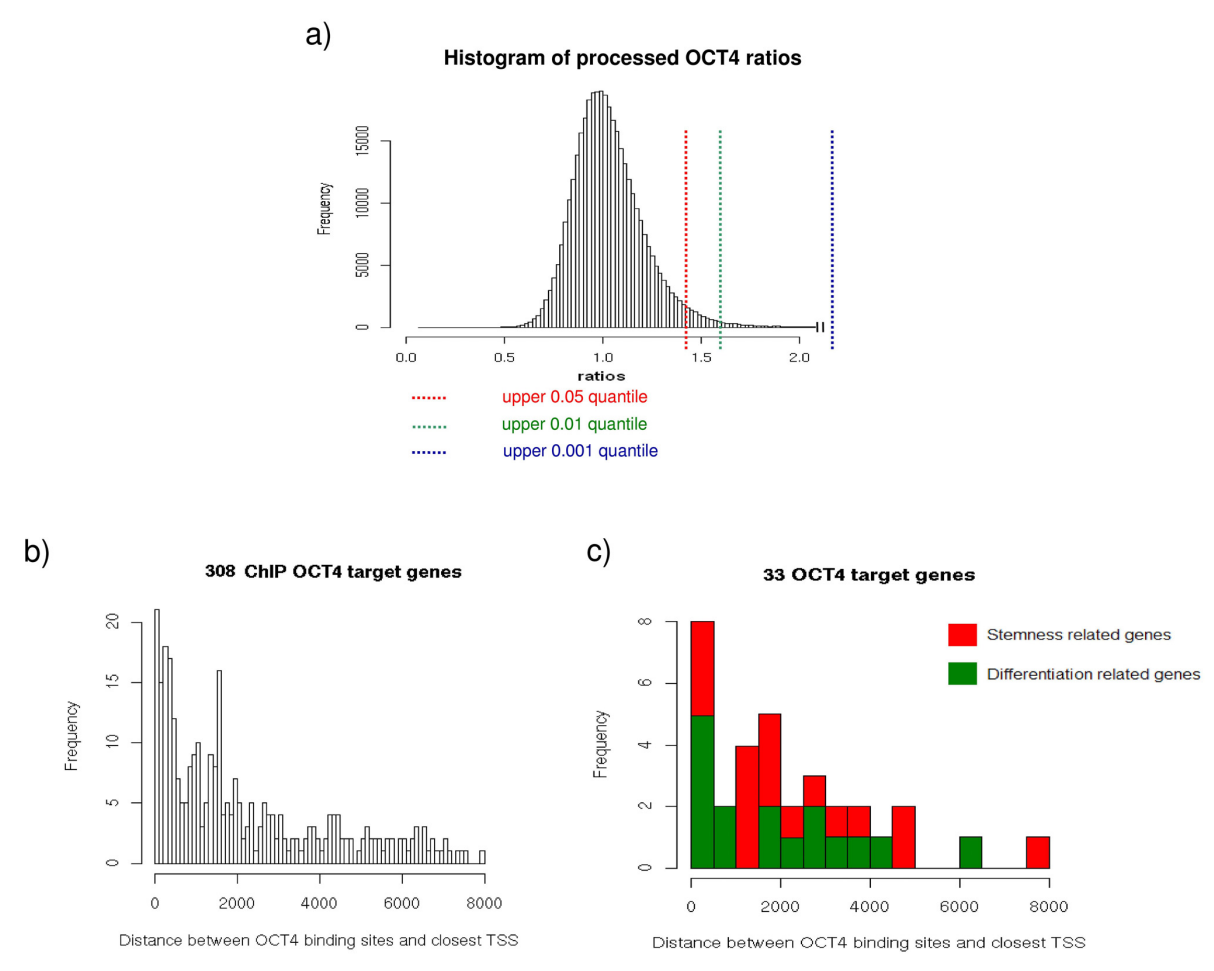
**Histogram of processed OCT4 ratios**. a) Histogram of quality controlled and normalized OCT4 ChIP-on-chip ratios for the individual probes. The blue line represents the threshold for the upper 0.001 quantile of the distribution (green line: upper 0.01 quantile and red line upper 0.05 quantile, respectively). b) Histogram of distances between OCT4 binding sites and closest TSS for all 308 validated OCT4 target genes, (c) Histogram of distances between OCT4 binding sites and closest TSS for the 33 isolated functional OCT4 target genes. The red fraction corresponds to stemness related genes, the green fraction to differentiation related genes.

We performed a re-analysis of the OCT4, SOX2 and NANOG ChIP-on-chip data from hESCs (NIH Code: WA09 cells) [12] including the mapping of the 60 mer oligonucleotide probes to an updated NCBI build (v36.1). In total, 230,068 oligonucleotides matched to their original position (+/-100 bp) whereas 141,270 probes were mapped more than 500 bp away from their original position. Processing of the uniquely-mapped probes includes background correction, normalization, fold-enrichment and peak identification and resulted in 308 potential OCT4 target genes (see Figure 1a for a histogram of OCT4 ChIP-on-chip ratios and Materials and Methods for a detailed description of the analysis). Figure 1b shows a histogram of the distances between binding sites and transcription start sites (TSSs) for the 308 direct OCT4 target genes. Most OCT4 binding sites (72.07%) are located less then 3 kb upstream of the TSS. 37.98% of all binding sites are located less then 1 kb upstream of the TSS and a second accumulation of binding sites is observed in the region between -1 kb to -2 kb with nearly a quarter (23.05%) of all binding sites.

Because protein-DNA binding events do not give information on the direction of the regulatory influence of the TF with respect to the transcription rate of its target genes, we complemented the results of the OCT4 ChIP-on-chip experiment with the results of the RNAi mediated OCT4 silencing in hESCs (H1 clone) performed by Babaie et al. [16]. Identifier mapping of the different chip platforms (Agilent oligochips and cDNA microarrays) resulted in 10,065 genes that were represented as cDNA clones on the microarray (see Materials and Methods) and that had promoter regions covered by the Agilent tiling arrays. From the originally published 623 OCT4 target genes [12], 472 were also represented on the cDNA microarray. From the 1,104 genes that show significantly altered expression 72 hours after the OCT4 knock down, 40 genes (<4%) were also identified as direct OCT4 target genes.

**Figure 2:**
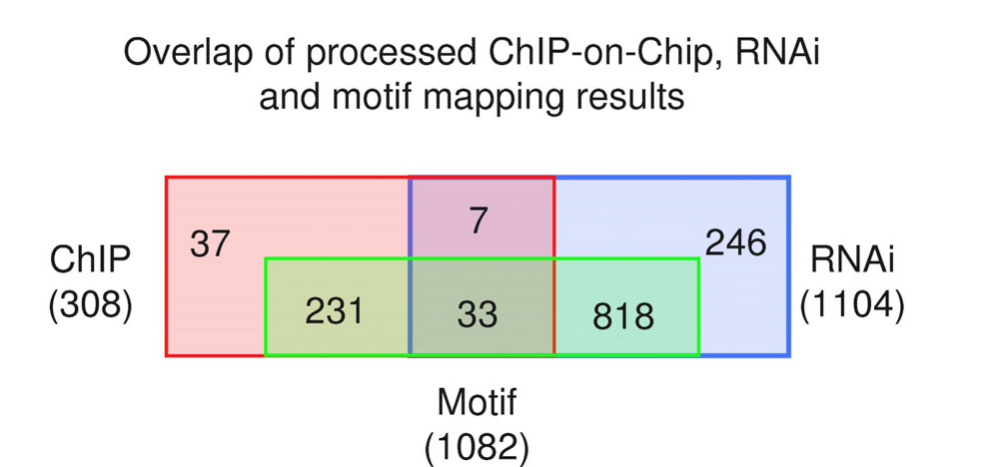
**Overlap of the individual studies**. Overlap of the re-analyzed OCT4 ChIP-on-chip experiment, the OCT4 RNAi experiment and the motif mapping results with the octamer and sox-oct joint motifs.

In order to obtain an even more stringent set of OCT4 target genes, we searched the promoter sequences of the targets for the occurrence of the known OCT4-related octamer and SOX-OCT joint motifs within a distance of 8 kb upstream of the respective TSSs (see Material and Methods). Even though we neglect information on binding events caused by OCT4-DNA interactions mediated by unknown cofactors and heterodimer complexes, our results reflect confirmed functional circuitries dependent on direct OCT4 and SOX-OCT binding. The combination of the three approaches resulted in a set of 33 genes (Figure 2). Nevertheless, it has to be mentioned that a motif could be mapped to the genomic environment of a ChIP-on-chip derived significant peak (distance of less then 1 kb) for only a third of these genes, whereas for the remaining genes the genomic position of the mapped motif is further away from the centre of the peaks.

The complete results of the individual studies (together with the results of the re-analyzed SOX2 and NANOG ChIP-on-chip experiments) are summarized in Additional file 1.

### Positional distribution of OCT4 transcription factor binding sites (TFBSs)

Figure 1c shows a histogram of the distances between binding sites and TSSs for the 33 functional OCT4 target genes. Similar to the histogram of all 308 validated direct OCT4 target genes (see Figure 1b), the majority of OCT4 binding sites (69.68%) are located less then 3 kb upstream of the TSS, 30.30% of all binding sites are located already less then 1 kb upstream of the TSS and a second accumulation of binding sites can be observed in the region between -1 kb to -2 kb with nearly a quarter (24.24%) of all binding sites. Interestingly, slight differences in the distribution of binding site distances to TSSs can be observed when the set of OCT4 target genes is split into functionally distinct subsets (see Figures 1c): 43.75% of the genes that are functionally connected to the process of differentiation (defined by negative regulation by OCT4) have the OCT4 binding site within the 1 kb upstream region of their TSS, whereas only 17.65% of the stemness related genes (defined by positive regulation by OCT4) have the OCT4 binding site within this region. On the other hand, 35.23% of the stemness related genes have an OCT4 binding site within the -1 kb to -2 kb region whereas only 12.5% of the differentiation related genes have the OCT4 binding site within this region. Therefore, it seems that differentiation related genes tend to have an OCT4 binding site closer to their TSS.

### OCT4 target genes

Among the 33 genes, several well-known targets of OCT4 can be found as well as genes whose regulatory interaction with OCT4 is less well-described. In general, OCT4 binds to and regulates diverse classes of genes encoding for example transcription factors (TGIF2, EOMES, FOXD3, GSC, TSC22D1, GATA6, OCT4, SOX2, NANOG, PAX6, CDX2, TCF4), transcriptional regulators (SSBP2), regulators of kinase, transferase, and catalytic activity (GAP43, TDGF1), members of the Wnt receptor signalling pathway (SFRP2, FRAT2, DKK1), and growth factors (FGF2, LEFTY2, TDGF1). A functional classification of the 33 OCT4 target genes is given in Additional file 2.

**Figure 3:**
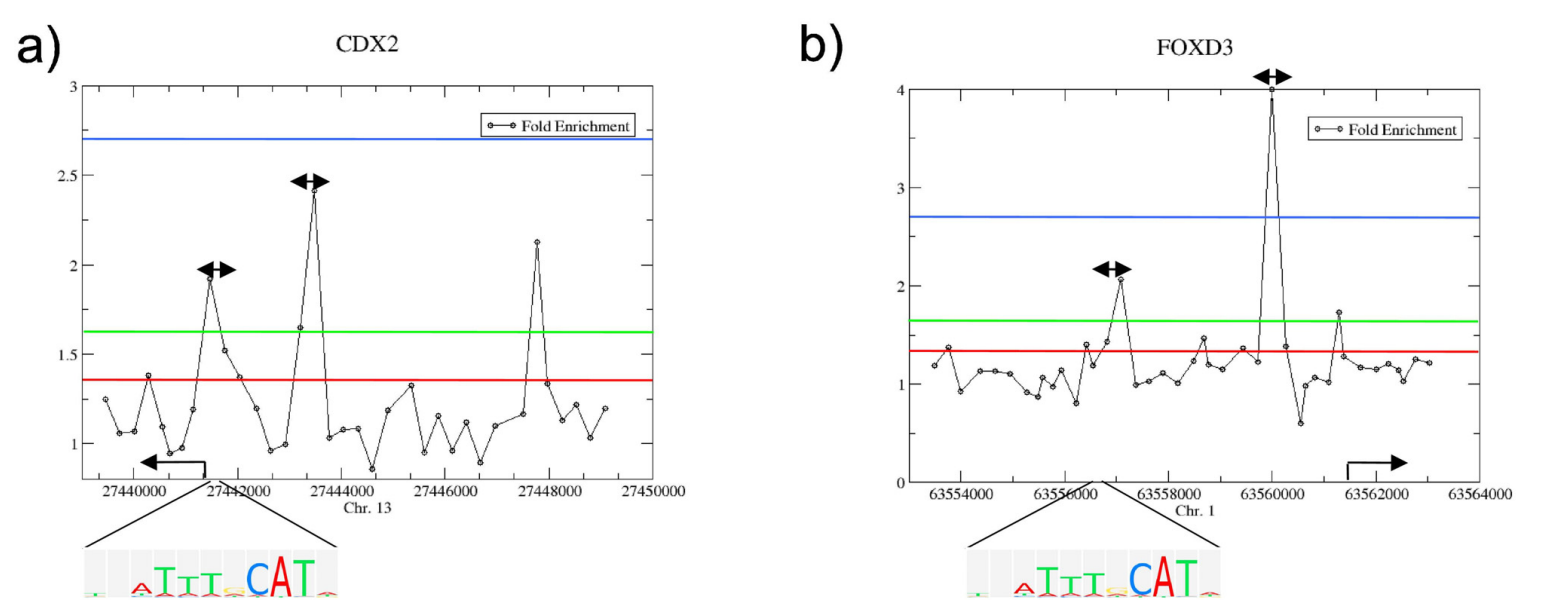
**Example peaks taken from the isolated set of OCT4 target genes**. Example peaks within the promoter regions of a) CDX2 and b) FOXD3, two of the isolated OCT4 target genes. The coloured lines refer to the thresholds corresponding to the quantiles given in Figure 1a. The motifs represent the octamer motif mapped to the respective promoter regions. The two-sided arrows illustrate the sub sequences taken for the *de novo *motif discovery.

As an example, Figure 3 illustrates ChIP-on-chip data results in the promoter regions of two target genes. Figure 3a shows two identified peaks located approximately 155 bp and 2027 bp upstream of the transcription start site of CDX2 (Caudal-type homeobox transcription factor 2). A binding event was identified for NANOG at the same genomic positions but not for SOX2. Additionally, the octamer motif was found approximately 233 bp upstream of the TSS. OCT4 negatively regulates the transcription of CDX2, as it is significantly up-regulated upon OCT4 knockdown [16]. This observation is in line with the function of CDX2 which encodes a protein that is important in a broad range of cellular functions such as trophoblast differentiation in human and mouse [17,18] to maintenance of the intestinal epithelial lining of both the small and large intestine [19]. Furthermore, it has been shown that Oct4 directly regulates the expression of Cdx2 in mouse embryonic stem cells [20,21].

As a second example, Figure 3b shows two identified peaks located approximately 1304 bp and 4212 bp upstream of the TSS of FOXD3 (Forkhead box protein D3). Binding events were identified for NANOG at the same genomic position but not for SOX2. The octamer motif was found approximately 4679 bp upstream of the TSS. Moreover, OCT4 has a positive regulatory influence on the transcription of FOXD3 as it is significantly down-regulated 72 hours after RNAi mediated OCT4 depletion [16]. This observation is consistent with the function of Foxd3 in mouse embryonic stem cells, as it is required for maintenance of progenitor cells in the inner cell mass and in the trophoblast [22,23]. Additionally, it has been shown that Foxd3 has an important role in repressing differentiation, promoting self-renewal, and maintaining survival of mouse ESCs [24].

Further evidence for the regulatory influence of OCT4 on the 33 target genes has been agglomerated from published experimental studies. Additional file 3 contains a glossary for the 33 core OCT4 target genes that summarizes further independent published experimental validations on the regulatory influence of OCT4 to its presented target genes.

### Integration of data enriches functional content of OCT4 target gene set

**Figure 4:**
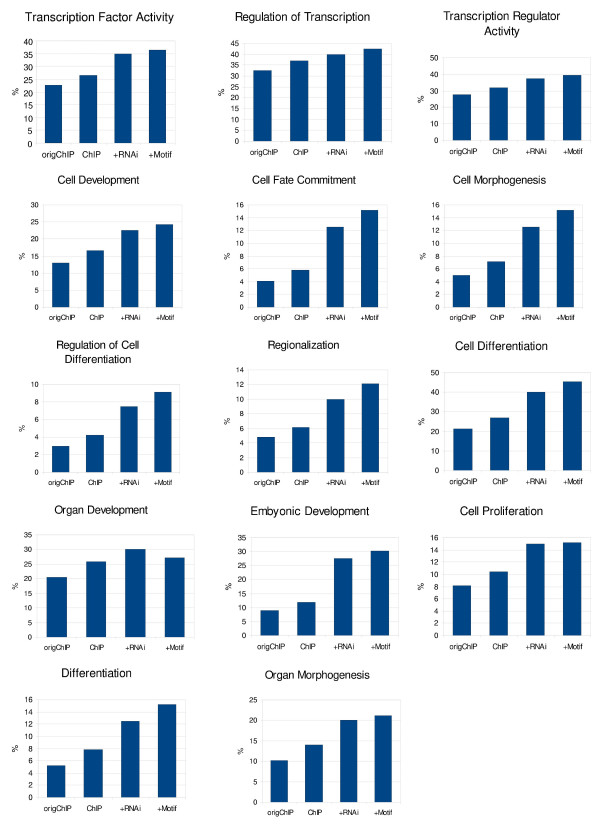
**Enrichment of functional information through integrated analysis**. Increase of functional information content of selected gene lists. As an example, the gene ontology term "GO:0003700 Transcription factor activity" is described in detail (from left to right): OCT4 target genes using the original ChIP-on-chip data (origChIP), using the re-analyzed OCT4 ChIP-on-chip data (ChIP), after integration of the results of the OCT4 RNAi experiment (+RNAi) and after integration of the motif mapping (+Motif). The corresponding absolute numbers of selected target genes are 122, 82, 14 and 12 (not indicated within the illustration).

Enrichment analysis [25] revealed that the functional information content of the gene set is accelerated (factors of 1.3 – 4.7) by integrating the results of the individual studies. Figure 4 shows the increase in the percentage of genes connected to gene ontology terms, for example "*GO:0003700: Transcription factor activity*", with respect to the original set of ChIP-on-chip targets (origChIP, 22.72%), the re-analyzed set of ChIP-on-chip target genes (ChIP, 26.62%), the additional filtering according to the RNAi experiment (+RNAi, 35%) and additional motif mapping (+Motif, 36.36%). The same trend can be observed with other GO terms such as "*GO:0030154: Cell differentiation"*, "*GO:0045165: Cell fate commitment"*, "*GO:0009790: Embryonic development"*, "*GO:0008283: Cell proliferation"*, and others (see Figure 4). Table 1 shows the top enriched gene ontology terms (see Additional file 2 for the complete results of the enrichment analysis).

**Table 1:** Enrichment analysis

**Term**	**Count**	**%**	**PValue**
GO:0007275~multicellular organismal development	18	54.55%	0.000000
GO:0003700~transcription factor activity	12	36.36%	0.000000
GO:0009653~anatomical structure morphogenesis	16	48.48%	0.000000
GO:0032502~developmental process	20	60.61%	0.000000
GO:0009790~embryonic development	10	30.30%	0.000000
GO:0048856~anatomical structure development	17	51.52%	0.000000
GO:0030528~transcription regulator activity	13	39.39%	0.000002
GO:0030154~cell differentiation	15	45.45%	0.000002
GO:0048869~cellular developmental process	15	45.45%	0.000002
GO:0007389~pattern specification process	6	18.18%	0.000010
GO:0032501~multicellular organismal process	19	57.58%	0.000019
GO:0045165~cell fate commitment	5	15.15%	0.000022
GO:0043565~sequence-specific DNA binding	8	24.24%	0.000029
GO:0003677~DNA binding	14	42.42%	0.000051
GO:0031323~regulation of cellular metabolic process	16	48.48%	0.000063
GO:0019222~regulation of metabolic process	16	48.48%	0.000095
GO:0009887~organ morphogenesis	7	21.21%	0.000105
GO:0031325~positive regulation of cellular metabolic process	7	21.21%	0.000107
GO:0009893~positive regulation of metabolic process	7	21.21%	0.000154
GO:0001824~blastocyst development	3	9.09%	0.000356
GO:0045449~regulation of transcription	14	42.42%	0.000413
GO:0019219~regulation of nucleobase, nucleoside, nucleotide and nucleic acid metabolic process	14	42.42%	0.000519
GO:0050789~regulation of biological process	19	57.58%	0.000533
GO:0065007~biological regulation	20	60.61%	0.000539
GO:0006350~transcription	14	42.42%	0.000597
GO:0009798~axis specification	3	9.09%	0.000640
GO:0050794~regulation of cellular process	18	54.55%	0.000754
GO:0010468~regulation of gene expression	14	42.42%	0.000756
GO:0006355~regulation of transcription, DNA-dependent	13	39.39%	0.000890

Top enriched gene ontology categories for the 33 OCT4 target genes as received by DAVID [25] (see Additional file 2 for the complete results).

### OCT4 core regulatory network

**Figure 5:**
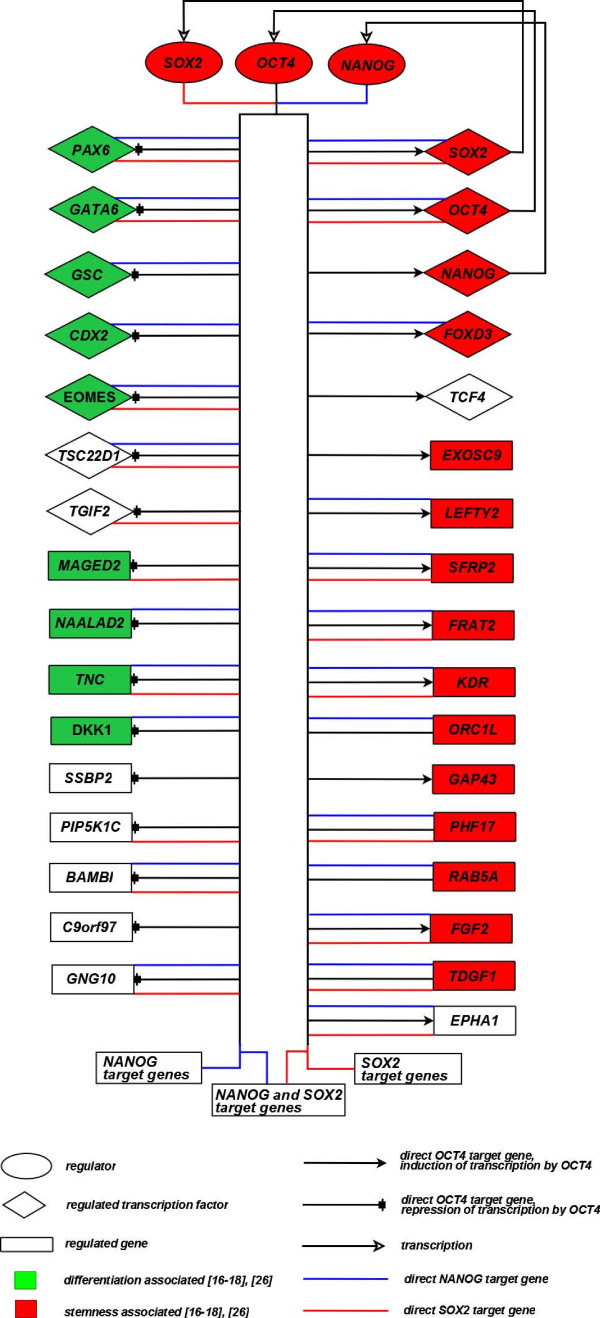
**OCT4 core regulatory network**. Core OCT4 transcriptional regulatory network identified by the integrative analysis of the re-analysed ChIP-on-Chip data, the OCT4 RNAi knock-down and the sequence-based octamer and sox-oct motif mapping. Green boxes represent genes associated with differentiation and red boxes indicate genes being specific for hESCs as annotated by several further public sources [16-18,26]. For white boxed genes no detailed annotation about differentiation or stemness characteristics was found by literature research. The network also incorporates the information on direct target genes from the re-analyzed SOX2 (red lines) and NANOG (blue lines) ChIP-on-chip experiments.

The resulting OCT4 core regulatory network, also incorporating the information on direct target genes from the re-analyzed SOX2 (red lines) and NANOG (blue lines) ChIP-on-chip experiments, is shown in Figure 5. The network distinguishes genes that are suppressed (left side) from those that are activated (right side) by OCT4. Among the 33 genes a high fraction is annotated with transcription factor activity (GO:0003700, indicated as rhombuses). Furthermore, a classification in hESCs specific genes (red boxes) and genes that are associated with the process of differentiation (green boxes) was performed by accessing several further public sources [16-18,26]. White boxed genes could not be annotated using these sources, but the information about up or down regulation after the OCT4 knock-down indicates, whether the respective gene is functional connected to the process of differentiation or to the maintenance of pluripotency.

An additional level of gene regulation has been added to this core OCT4 target network by further literature and database mining (Additional file 4). This additional figure shows the core network extended by known up- and downstream target genes of the respective TFs as given by TRANSFAC [19] and by another published work [27].

Further interactions of the OCT4 target genes were revealed using the ConsensusPathDB [28], a database that integrates the content of 12 different interaction databases with heterogeneous foci. As an example, Additional file 5 shows known interaction partners of CDX2. Among these interactions, interestingly, a physical interaction is observed between CDX2 and PAX6, that is another important differentiation associated TF included in the presented set of OCT4 target genes. The core OCT4 network is represented in SBML format (Additional file 6) which can be used for further studies, e.g, mathematical modelling.

### *De novo *motif discovery

**Figure 6:**
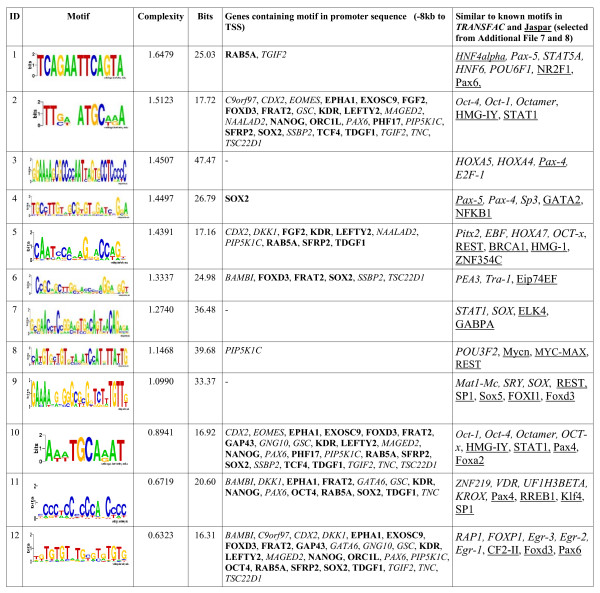
***De novo *motif discovery**. 12 motifs (second column) identified by a *de novo *motif discovery approach, sorted by their complexity value derived from the di-mer distribution (third column). The second and tenth motifs have a high similarity with known OCT4 related motifs. The fifth column lists the genes from the network that contain the motif within their promoter regions. Genes being suppressed by OCT4 are stated italic, genes being activated by OCT4 are stated bold. The last column shows selected similarity matched motifs from TRANSFAC (stated italic, [19]) and Jaspar (stated underlined, [31]) as received by STAMP [32] (see Additional files 8 and 9 for the full results).

Transcription factor co-localizations targeted by multiple transcription factors are sites that integrate the external signalling pathways to the transcriptional regulatory circuitry governed by OCT4, SOX2, and NANOG and these sites may serve as focal points for the assembly of further regulatory nucleoprotein complexes [29]. In order to test for further regulatory co-factors of OCT4, we performed a *de novo *motif discovery analysis based on specific promoter regions of the 308 direct OCT4 target genes derived from the re-analysis of the ChIP-on-chip data (sub-sequences of length 200 bp around the identified peaks; such selected regions are highlighted as arrows in Figure 3). The selected sub-sequences were repeat-masked [30] and used as input for several *de novo *motif discovery algorithms (see Material and Methods). We identified 12 unique sequence motifs of higher quality (Figure 6 and Additional file 7). These motifs were compared against two existing databases of known motifs (TRANSFAC [19] and JASPAR [31]) using the STAMP tool [32] (for the complete results see Additional files 8 and 9). Motifs similar to the octamer and sox-oct joint motifs were discovered. Additionally, sequence motifs were identified that are potentially recognized by factors involved in maintaining pluripotency and development. For the 33 core OCT4 target genes, Figure 6 lists the individual genes that contain the discovered motifs within their promoter regions.

Interestingly, we identified a motif similar to the binding site of MYC-MAX (see Figure 6), a heterodimer of the transcription factors C-MYC and MAX, a pre-requisite for C-MYC transcriptional activity that leads to cell growth and proliferation [33,34]. C-MYC has been utilized in a set of four transcription factors for deriving iPS cells from somatic cells [1,2]. The C-MYC related motif was identified in the promoter region of PIP5K1C which encodes PIPKI gamma of the phophatidylinsitol pathway [35]. C-MYC is even further connected to the presented OCT4 regulatory network as it is a direct target of TCF4 (Transcription factor 7-like 2) whose transcription is enhanced by OCT4 function and which is a CTNNB1 binding protein that regulates Wnt signalling, cell cycle, and cell proliferation [19]. Moreover, C-MYC is a direct target of E2F/DP, a complex essential for cell proliferation [36]. Additionally, E2F/DP directly binds to ORC1L (Origin recognition complex subunit 1-like), another member of the network which encodes for a chromatin binding protein that plays a role in DNA replication initiation and mitotic cell cycle, induces caspase activation, cell surface exposure of phosphatidylserine, and DNA fragmentation during apoptosis [19]. A motif similar to the binding site of E2F-1 was obtained by the *de novo *motif discovery. Based on an analogous *de novo *motif discovery performed on the sub-sequences of validated peak regions taken from the promoter regions of the 33 core OCT4 target genes only, a motif similar to the binding site of E2F was obtained (consensus sequence *GSmkGs*) and could be mapped to the promoter regions of ORC1L and further OCT4 target genes (data not shown).

## Discussion

We have performed an integrative study of complementary experimental techniques in order to identify the core gene regulatory network of OCT4 in the context of maintaining pluripotency and preventing differentiation along the trophoblast lineage. This network represents a rather conservative selection since we have chosen only genes with high evidence that showed significant results in ChIP-on-chip analysis, RNAi analysis and promoter sequence analysis.

Figure 2 illustrates the limited overlap between the different technologies. For example, only 40 genes intersect between the ChIP-on-chip (12%) and the RNAi experiments (4%). This observation is in line with the results of a similar approach comparing the overlap of altered gene expression after Oct4 silencing and TF binding in mouse ESCs (<9% overlap) [20]. Genes that show altered gene expression but no binding site may be regulated by an inter-dependent network, where loss of expression of one factor ultimately leads to the suppression of the others [16]. Additionally, the RNAi targets relate also to downstream effects independent of direct protein-DNA binding of OCT4 which explains the higher number of RNAi targets (1,104) compared to ChIP-on-chip targets (308). Alternatively, TF binding may not be limited to the promoter region interrogated by the tiling arrays. On the other hand, genes having OCT4 binding sites but do not show altered expression may be regulated by a more complex system of OCT4 co-factors, epigenetic modifications like de-/methylation of CpG di-nucleotides within promoter regions or simply at later time points during differentiation into one of the three germ layers or into the trophectoderm lineage. Hence, independent validations such as the accessed knockdown experiment are critical in distinguishing functional from non-functional circuitries [20].

Although the presented network is rather conservative and potentially neglects genes regulated by OCT4 together with unknown interaction partners, it represents the functional regulatory circuitry of direct OCT4 target genes in hESCs as deduced by the available data. It is a well-known problem of both ChIP-on-chip experiments and motif prediction analyses to generate a large number of false positives. Additionally, RNAi experiments do not only reveal direct but – to a much higher extent – indirect targets. Thus, having a rather conservative process for identifying OCT4 target genes has the benefit of narrowing down this large number of false positives. An indicator of this is the fact that the integration of the different experiments purifies and enriches functional content of the resulting targets in all investigated functional classes by factors of 1.5 – 4 as is shown in Figure 4.

Recent studies report *OCT4*/*Oct4 *expression in the adult, most frequently in the bone marrow of both humans and mice, particularly in hematopoietic and mesenchymal stem cells as well as in various sub-populations of multipotent progenitors [37]. It has been suggested that *Oct4 *may not only be crucial for the maintenance of pluripotency in embryonic cells but may also play an important role for the self-renewal of somatic stem cells [37]. However, Lengner et al. [37] have shown that *Oct4*, even if expressed at low levels in somatic cells, is dispensable for the self-renewal of somatic stem cells, and for the regeneration of tissue in the adult, and is only rarely activated in somatic tumors. Based on these observations, we do not consider *OCT4*/*Oct4 *to be a key player for transcriptional regulation of pluripotency in either mesenchymal stem cells and other adult stem cells. The identified core regulatory network of OCT4 was created in the context of human embryonic stem cells for maintaining pluripotency and preventing differentiation along the trophoblast lineage.

Our *de novo *motif discovery approach did not only reveal known OCT4 binding sites but also motifs similar to binding sites recognized by regulators that are known to interact with components of the OCT4 regulatory network as well as genes that may have important functions as downstream effectors of OCT4 but not yet described. Besides the co-factors presented above, a predominantly occurring motif is similar to a binding site recognized by Sp1 (Specificity protein 1), a transcription regulator that plays a role in TGF beta induced cell migration and mesenchymal transition, regulates angiogenesis, heart contraction, and aberrant expression is associated with several types of cancer. Yang et al. proposed that Sp1 or Sp3 play a critical role in controlling the transcriptional activity of OCT4 by direct binding and an overexpression study showed that Sp1 positively regulates OCT4 promoter activity [38]. The Sp1 motif was identified within the promoter regions of OCT4 and of other OCT4 target genes (see Figure 6). Sp1 is closely connected to the network as it binds to FGF2, C-MYC, HOXB7, Spp1 (the latter two genes are upregulated by Sp1), and interacts with Egr-1. Moreover, Sp1 interacts with CP2A, a TF which in turn regulates PAX6 [19] (not indicated in the extended network), a transcription factor which is a member of the differentiation related OCT4 target genes. From the mouse model it is known that Sp1 binds to Foxa1 and Cdx2. Egr-1 (Early growth response 1) is a transcription factor that acts in apoptosis, angiogenesis, cell differentiation, regulates TNF production, cell proliferation and adhesion and aberrant expression of the gene is associated with several types of cancer. HOXB7 (Homeo box B7) is a transcriptional activator and functions in DNA double strand break repair by nonhomologous end joining. Both, Egr-1 and HOXB7 bind to the promoter region of FGF2 and a motif similar to the binding site of Egr-1 was obtained (see Figure 6).

As another example, the *de novo *motif discovery identified a binding site similar to a motif recognized by STAT1 (Signal transducer and activator of transcription 1), a gene that mediates DNA replication, cell proliferation, apoptosis, and cell cycle regulation. It is known that STAT1 binds to C-MYC and is upregulated by Sp1. Several of the OCT4 target genes show a putative STAT1 binding site within their promoter region (see Figure 6).

HNF4A (Hepatocyte nuclear factor 4 alpha) has a known binding site similar to one of the obtained motifs. It is a transcription factor that inhibits GH1 induced STAT5 and JAK2 phosphorylation and functions in hepatocyte differentiation and blood coagulation. HNF4A expression is upregulated by Sp1 and is a target of GATA6, a transcription factor which is a member of the differentiation related OCT4 target genes. RAB5A and TGIF2 show a putative HNF4A binding site within their promoter region (see Figure 6).

PAX4 (Paired box gene 4) is a putative RNA polymerase II transcription factor that acts in positive regulation of cell proliferation and motifs similar to the known binding site for PAX4 were obtained. PAX4 itself has a binding site for HNF4A which is a downstream target of GATA6 [19]. There are putative PAX4 binding sites within the promoter regions of several OCT4 target genes (see Figure 6).

The computed OCT4 core regulatory network can be utilized in multiple ways. Well-characterized OCT4 target genes will help in extending the OCT4 network by suggesting further experimental work. The relatively high proportion of TFs in the OCT4 target set can be used for further inhibition studies or protein-DNA binding experiments. This leads to an extended radius of the network. For example, Additional file 4 shows that OCT4 has a positive regulatory effect on FGF2. FGF2 re-stimulation experiments performed by Greber et al. in hESCs revealed BMP4 as a downstream target of FGF2 signaling [27]. BMP4 expression was activated upon OCT4 knockdown in the original experiment as well, so both experiments consistently confirm that BMP4 is a negatively regulated downtream target of OCT4. Such an extended network and even the constructed core regulatory network will ultimately help in the study of stemness and early embryogenesis. Figure 4 shows functional enrichment for "embryonic development" that is increasing from 8%–30% with the integrative approach.

Finally, the identification of targets and co-factors of OCT4 might help in the design of iPS reprogramming protocols that use different TFs in order to generate and monitor cell status. C-MYC has already been successfully applied within a set of TFs for generating iPS cells through reprogramming. Figure 6 gives a guided hint for testing a variety of these co-factors.

## Conclusion

The OCT4 dependent functional transcriptional regulatory network important in the analysis of human stem cell characteristics and cellular differentiation was reconstructed using an integrative approach. Functional information is largely enriched using an overlay of different experimental results. The *de novo *motif discovery points out several well known regulators closely connected to the network as well as less described potential downstream regulators of pluripotency and differentiation.

## Methods

### ChIP-on-chip data re-analysis

For identifying DNA regions occupied by OCT4, SOX2 and NANOG, Boyer et al. [12] performed two ChIP-on-chip experiments for each of the three transcription factors. They utilized a set of ten promoter arrays containing in total 399,309 60 mer oligonucleotides, and the design of the oligonucleotides was based on the NCBI build 35 of the human genome. As an update, the oligonucleotides were mapped [39] to the NCBI build 36.1 (hg18, Mar. 2006) and the updated allocation of 373,181 uniquely matched oligonucleotides to their genomic positions served as reference for the subsequent peak-finding. For all uniquely mapped oligonucleotides, the available raw expression data was background corrected, array-wise quantile normalized and replicates were normalized between arrays by applying Bioconductor's limma package [40]. For each oligonucleotide, a fold-enrichment was calculated by dividing the averaged signal intensities of the immunoprecipitated replicates by the averaged signal intensities of the whole-genome replicates. A histogram of the ratios from the OCT4 replicates is given in Figure 1. Potential binding events were defined as A) one oligonucleotide having a ratio within the upper 0.001 quantile of the total ratio distribution or B) two neighbouring oligonucleotides within a window of 1 kb where one oligonucleotide has a ratio within the upper 0.01 quantile and the other one has a ratio within the upper 0.05 quantile of the total ratio distribution. These two filters were defined by considering possible binding events between sonicated DNA fragments of an estimated averaged length of 550 bp and the oligonucleotides on the arrays with respect to the density of the newly assigned oligonucleotides relative to the genomic promoter regions. All identified peaks were connected to the closest TSS, if one exists within a distance of 10 kb. Genomic positions of TSSs were based on Ensembl [41] and were downloaded via Biomart [42]. By this procedure, 308 genes of the original 623 identified OCT4 targets could be identified.

### Motif mapping of promoter sequences

The promoter regions of the combined targets taken from the validated ChIP-on-chip and from the RNAi experiments were tested for the occurrence of the octamer and the SOX-OCT joint motifs. Position-specific count matrices were retrieved from TRANSFAC database v12.1 [19] for the octamer (TRANSFAC id *V$OCT_Q6*) and the SOX-OCT joined motifs (TRANSFAC ids *V$OCT4_02 *and *V$OCT4_01*). These were converted to regularized and scaled position-specific scoring matrices (PSSMs) using an in-house implementation of the method of Rahmann et al. [43]. For each of the genes, -8 Kb to +2 Kb of the TSS were retrieved from the ENSEMBL (version 47) database and scanned with the PSSMs for the maximum scoring hit on each sequence. To focus on the upstream promoter region and have motifs of reasonable quality, only the subset of maximum scoring hits which lay in the upstream region of -8 Kb and scored above 70% of the maximum attainable score for a given PSSM were recorded.

### Enrichment analysis

Enrichment analysis was conducted with the DAVID platform [25]. Official gene symbols were used as input, the *Homo Sapiens *species was selected as background and DAVID was executed with default parameter settings.

### *De novo *motif discovery

In order to test for further regulatory co-factors of OCT4, we performed a *de novo *motif discovery analysis based on specific promoter regions of the 308 direct OCT4 target genes derived from the re-analysis of the ChIP-on-Chip data. By taking the genomic positions of the identified peaks as a reference (that is the position of an oligonucleotide or the centre of oligonucleotides detecting a peak, respectively), we assembled the sub-sequences of length 200 bp around the peaks (bandwidth of length 100 bp, as an example see arrows in Figure 3). The selected sub-sequences were repeat masked [30] and used as input for the TAMO package, a *de novo *motif discovery framework [44] that incorporates AlignACE [45], MDScan [46] and MEME [47]. The motif discovery was performed following the given sample code except the clustering module. Additionally, we used the Gibbs Motif Sampler [48] implementation of the CisGenome [49] framework with default parameter settings. All obtained motifs were compared to each other by applying the *minaligndiff *function of the TAMO distribution and when motifs occur with an alignment difference < 0.2, only the motif with the highest Bit score is further considered. Motifs with Bit score < 15 were discarded. Secondly, we computed entropy of the di-mer distribution of the motif sequence as a measure for the motif complexity. Motifs with complexity score < 0.6 were discarded. The remaining 12 unique motifs were sorted by their complexity value and are shown in Figure 6.

### Database matching of discovered motifs

The discovered motifs were compared against two databases of known motifs using the STAMP tool [32]. Motifs were compared against the TRANSFAC (v11.3) [19] and JASPAR (v3) [31] databases using the recommended default parameter settings.

## Abbreviations

iPS: induced pluripotent stem cells; TF: transcription factor; ESCs: embryonic stem cells; TSSs: transcription start sites; TFBS: transcription factor binding sites; PSSMs: position-specific scoring matrices.

## Authors' contributions

LC carried out the ChIP-on-chip data re-analysis, the analysis of the positional distribution of OCT4 transcription factor binding sites, the integration of the individual methods, created the OCT4 core regulatory network, performed the functional enrichment analyses of the OCT4 target genes, the *de novo *motif discovery and the database matching of discovered motifs and drafted the manuscript. ASB performed the motif mapping to the promoter sequences. MV and HL participated in the design of the study and performed manuscript editing. JA and RH generated and analysed the RNAi data, conceived the study, participated in its design and co-ordination and helped to draft the manuscript. All authors have read and approved the final manuscript.

## Supplementary Material

Additional file 1 **Summary of the results of the individual studies**. The table includes all genes identified by the OCT4, SOX2, and NANOG ChIP-on-chip experiments [12] and by the RNAi mediated silencing of OCT4 function with subsequent microarray analysis [16]. The yellow column shows the influence of OCT4 to the transcription rate of its target genes (0 = no influence, 1 = OCT4 enhances transcription, -1 = OCT4 suppresses transcription). The green columns indicate OCT4 binding to its target genes as published by Boyer et al. and as identified via the presented data validation; the numbers indicate the distance between the TSS of the appropriate gene and the closest peak. The red and blue columns are constructed analogue but show the results for the SOX2 and NANOG experiments. The light brown columns represent the results of the octamer and SOX-OCT joint motif mapping (0 = promoter region contains the motif, 1 = promoter region does not contain the motif, n.a. = promoter region was not tested; we tested only the promoter regions of OCT4 target genes as identified by the re-analyzed ChIP-on-chip and by the RNAi experiments). Click here for file

Additional file 2 **Functional Annotation and Enrichment Analysis**. The table includes a list of the 33 OCT4 target genes together with their full gene names, a list of enriched functional groups and the results of the functional annotation clustering as received by Dennis et al. [25]. Click here for file

Additional file 3 **Glossary of target genes**. A glossary for the 33 core OCT4 target genes that summarizes further independent published experimental validations on the regulatory influence of OCT4 to its presented target genes. Click here for file

Additional file 4 **Extended network**. An additional level of gene regulation has been added to the core OCT4 target network (Figure 5) by further literature and database mining. This additional figure shows the core network extended by known up- and downstream target genes of the respective TFs as given by TRANSFAC [19] and by another published work [27]. Click here for file

Additional file 5 **CDX2 subnetwork from ConsensusPathDB**. The image illustrates the CDX2 centred sub-network as received from the ConsensusPathDB [28] and points out several known downstream target genes as well as a physical interaction between CDX2 and PAX6, another important differentiation associated TF included in the presented set of OCT4 target genes. Click here for file

Additional file 6 **OCT4 network as SBML**. The text file contains the core OCT4 network in SBML format. Click here for file

Additional file 7 **Discovered motifs as probability matrices**. The text file includes the 12 identified motifs as probability matrices as received from the TAMO package [44]. Click here for file

Additional file 8 **Motif database matching results- TRANSFAC**. The pdf file contains the results from comparing the 12 discovered motifs to the TRANSFAC database (v11.3, [19]) using the STAMP tool [32]. Click here for file

Additional file 9 **Motif database matching results- JASPAR**. The pdf file contains the results from comparing the 12 discovered motifs to the JASPAR database (v3, [31]) using the STAMP tool [32]. Click here for file
