# Supplementary material for: In silico identification of a core regulatory network of OCT4 in human embryonic stem cells using an integrated approach
Source: BMC Genomics. 2009 Jul 15;10:314. doi: 10.1186/1471-2164-10-314 (PMC2714862; doi:10.1186/1471-2164-10-314)
Supplement: Additional file 3 — Glossary of target genes. A glossary for the 33 core OCT4 target genes that summarizes further independent published experimental validations on the regulatory influence of OCT4 to its presented target genes. [file 1471-2164-10-314-S3.doc]

| Gene Name | Direct binding | Transcriptional regulation | Cell type |
| --- | --- | --- | --- |
| BAMBI | - | [1] | mESCs |
| C9orf97 | - | - | - |
| CDX2 | [2] [3] | [4] [5] [2] [6] [1] [7] | mESCs, hESCs |
| DKK1 | [8] [9] [2] [10] | [5] [1] [7] | mESCs, hECCs |
| EOMES | [8] [2] | [5] [2] [1] | mESCs, hECCs |
| EPHA1 | [8] | [11] [1] | mESCs, hECCs |
| EXOSC9 | [10] | [1] | mESCs |
| FGF2 | - | [11] | hECCs |
| FOXD3 | - | [2] [1] | mESCs |
| FRAT2 | - | [11] [1] [7] | mESCs, hECCs |
| GAP43 | [8] | [2] [11] [1] | mESCs, hECCs |
| GATA6 | [10] | [1] [7] | mESCs |
| GNG10 | - | [2] | mESCs |
| GSC | [3] [10] | [7] | mESCs |
| KDR | - | - | - |
| LEFTY2 | [9] [10] | [2] [1] | mESCs |
| MAGED2 | - | [2] [11] [1] | mESCs, hECCs |
| NAALAD2 | [8] | [11] | hECCs |
| NANOG | [8] [9] [12] [13] [2] [3] [10] | [13] [2] [11] [6] [1] [7] | mESCs, hESCs, hECCs |
| OCT4 | [8] [14] [2] [15] [3] [10] | [5] [2] [15] [11] | mESCs, hECCs, hESCs |
| ORC1L | [9] | [2] [11] [1] [7] | mESCs, hECCs |
| PAX6 | [8] [9] [3] [10] | [5] [2] [1] [7] | mESCs, hECCs |
| PHF17 | - | [7] | mESCs |
| PIP5K1C | [8] | [11] | hECCs |
| RAB5A | [10] | [5] [2] [7] | mESCs |
| SFRP2 | [9] [10] | [5] [2] [11] [1] | mESCs, hECCs |
| SOX2 | [8] [9] [2] [3] [10] | [4] [2] [1] [7] | mESCs, hECCs |
| SSBP2 | - | [11] [1] [7] | mESCs, hECCs |
| TCF4 | [8] | [2] [1] [7] | mESCs, hECCs |
| TDGF1 | [8] [9] [2] [3] | [5] [2] [11] [1] [7] | mESCs, hECCs |
| TGIF2 | [8] [10] | [1] | mESCs, hECCs |
| TNC | [8] | [11] [7] | mESCs, hECCs |
| TSC22D1 | [10] | [1] | mESCs |

**Glossary**

The glossary summarizes further independent published experimental validations on the regulatory influence of OCT4 to its presented 33 core target genes (mESCs: mouse embryonic stem cells, hESCs: human embryonic stem cells, hECCs: human embryonic carcinoma cells).

1. Sharov, A.A., S. Masui, L.V. Sharova, Y. Piao, K. Aiba, R. Matoba, L. Xin, H. Niwa, and M.S. Ko, **Identification of Pou5f1, Sox2, and Nanog downstream target genes with statistical confidence by applying a novel algorithm to time course microarray and genome-wide chromatin immunoprecipitation data***.* *BMC Genomics*, 2008. **9**: p. 269.

2. Loh, Y.H., Q. Wu, J.L. Chew, V.B. Vega, W. Zhang, X. Chen, G. Bourque, J. George, B. Leong, J. Liu, K.Y. Wong, K.W. Sung, C.W. Lee, X.D. Zhao, K.P. Chiu, L. Lipovich, V.A. Kuznetsov, P. Robson, L.W. Stanton, C.L. Wei, Y. Ruan, B. Lim, and H.H. Ng, **The Oct4 and Nanog transcription network regulates pluripotency in mouse embryonic stem cells***.* *Nat Genet*, 2006. **38**(4): p. 431-40.

3. Mathur, D., T.W. Danford, L.A. Boyer, R.A. Young, D.K. Gifford, and R. Jaenisch, **Analysis of the mouse embryonic stem cell regulatory networks obtained by ChIP-chip and ChIP-PET***.* *Genome Biol*, 2008. **9**(8): p. R126.

4. Niwa, H., J. Miyazaki, and A.G. Smith, **Quantitative expression of Oct-3/4 defines differentiation, dedifferentiation or self-renewal of ES cells***.* *Nat Genet*, 2000. **24**(4): p. 372-6.

5. Ivanova, N., R. Dobrin, R. Lu, I. Kotenko, J. Levorse, C. DeCoste, X. Schafer, Y. Lun, and I.R. Lemischka, **Dissecting self-renewal in stem cells with RNA interference***.* *Nature*, 2006. **442**(7102): p. 533-8.

6. Zafarana, G., S.R. Avery, K. Avery, H.D. Moore, and P.W. Andrews, **Specific Knockdown of OCT4 in Human Embryonic Stem Cells by Inducible Short Hairpin RNA Interference***.* *Stem Cells*, 2009. **27**(4): p. 776-782.

7. Matoba, R., H. Niwa, S. Masui, S. Ohtsuka, M.G. Carter, A.A. Sharov, and M.S. Ko, **Dissecting Oct3/4-regulated gene networks in embryonic stem cells by expression profiling***.* *PLoS ONE*, 2006. **1**: p. e26.

8. Jin, V.X., H. O'Geen, S. Iyengar, R. Green, and P.J. Farnham, **Identification of an OCT4 and SRY regulatory module using integrated computational and experimental genomics approaches***.* *Genome Res*, 2007. **17**(6): p. 807-17.

9. Kim, J., J. Chu, X. Shen, J. Wang, and S.H. Orkin, **An extended transcriptional network for pluripotency of embryonic stem cells***.* *Cell*, 2008. **132**(6): p. 1049-61.

10. Chen, X., H. Xu, P. Yuan, F. Fang, M. Huss, V.B. Vega, E. Wong, Y.L. Orlov, W. Zhang, J. Jiang, Y.H. Loh, H.C. Yeo, Z.X. Yeo, V. Narang, K.R. Govindarajan, B. Leong, A. Shahab, Y. Ruan, G. Bourque, W.K. Sung, N.D. Clarke, C.L. Wei, and H.H. Ng, **Integration of external signaling pathways with the core transcriptional network in embryonic stem cells***.* *Cell*, 2008. **133**(6): p. 1106-17.

11. Greber, B., H. Lehrach, and J. Adjaye, **Silencing of core transcription factors in human EC cells highlights the importance of autocrine FGF signaling for self-renewal***.* *BMC Dev Biol*, 2007. **7**: p. 46.

12. Kuroda, T., M. Tada, H. Kubota, H. Kimura, S.Y. Hatano, H. Suemori, N. Nakatsuji, and T. Tada, **Octamer and Sox elements are required for transcriptional cis regulation of Nanog gene expression***.* *Mol Cell Biol*, 2005. **25**(6): p. 2475-85.

13. Rodda, D.J., J.L. Chew, L.H. Lim, Y.H. Loh, B. Wang, H.H. Ng, and P. Robson, **Transcriptional regulation of nanog by OCT4 and SOX2***.* *J Biol Chem*, 2005. **280**(26): p. 24731-7.

14. Okumura-Nakanishi, S., M. Saito, H. Niwa, and F. Ishikawa, **Oct-3/4 and Sox2 regulate Oct-3/4 gene in embryonic stem cells***.* *J Biol Chem*, 2005. **280**(7): p. 5307-17.

15. Chew, J.L., Y.H. Loh, W. Zhang, X. Chen, W.L. Tam, L.S. Yeap, P. Li, Y.S. Ang, B. Lim, P. Robson, and H.H. Ng, **Reciprocal transcriptional regulation of Pou5f1 and Sox2 via the Oct4/Sox2 complex in embryonic stem cells***.* *Mol Cell Biol*, 2005. **25**(14): p. 6031-46.
