# Supplementary figures and images for: In silico identification of a core regulatory network of OCT4 in human embryonic stem cells using an integrated approach
Source: BMC Genomics. 2009 Jul 15;10:314. doi: 10.1186/1471-2164-10-314 (PMC2714862; doi:10.1186/1471-2164-10-314)

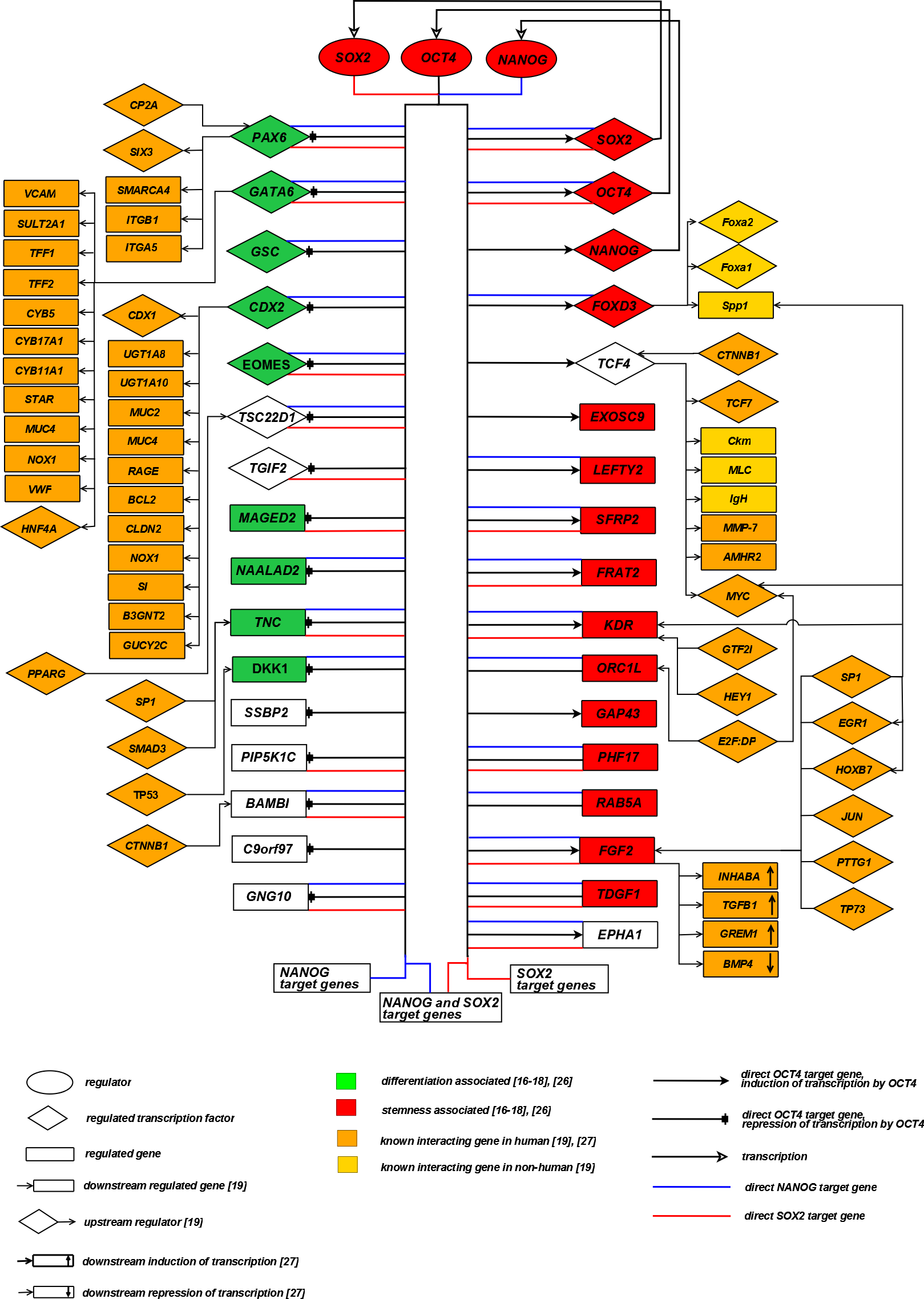

Supplement: Additional file 4 — Extended network. An additional level of gene regulation has been added to the core OCT4 target network (Figure 5) by further literature and database mining. This additional figure shows the core network extended by known up- and downstream target genes of the respective TFs as given by TRANSFAC [19] and by another published work [27]. [file 1471-2164-10-314-S4.png]

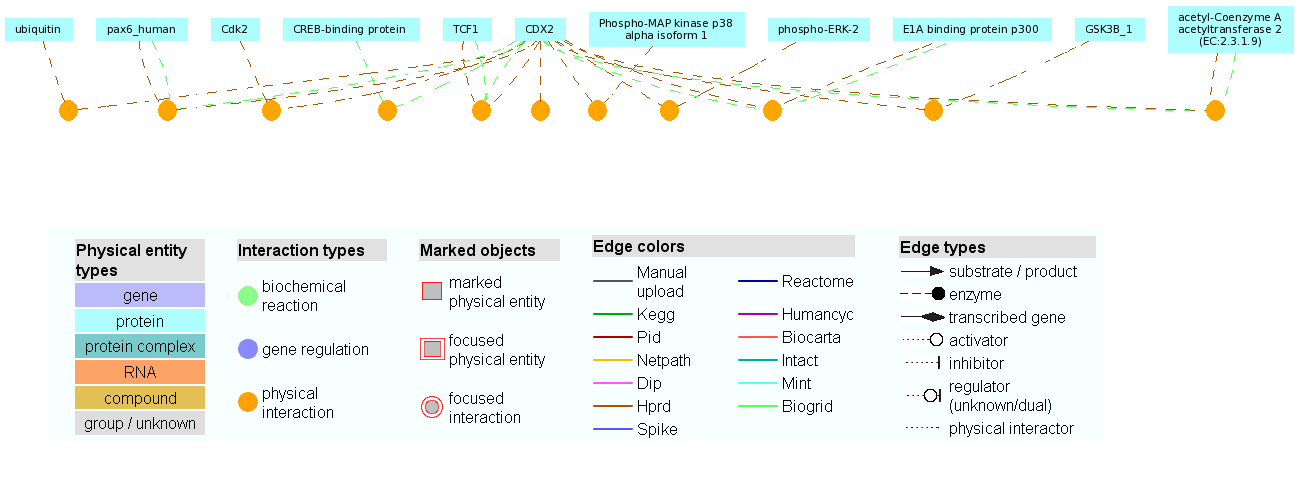

Supplement: Additional file 5 — CDX2 subnetwork from ConsensusPathDB. The image illustrates the CDX2 centred sub-network as received from the ConsensusPathDB [28] and points out several known downstream target genes as well as a physical interaction between CDX2 and PAX6, another important differentiation associated TF included in the presented set of OCT4 target genes. [file 1471-2164-10-314-S5.png]
