# Supplementary material for: In silico identification of a core regulatory network of OCT4 in human embryonic stem cells using an integrated approach
Source: BMC Genomics. 2009 Jul 15;10:314. doi: 10.1186/1471-2164-10-314 (PMC2714862; doi:10.1186/1471-2164-10-314)
Supplement: Additional file 8 — Motif database matching results- TRANSFAC. The pdf file contains the results from comparing the 12 discovered motifs to the TRANSFAC database (v11.3, [19]) using the STAMP tool [32]. [file 1471-2164-10-314-S8.pdf]

Jump to: Multiple Alignment Motif Tree Motif Matching
Input file: 12 motifs loaded
Settings: Metric=PCC, Alignment=SWU, Gap-open=1000, Gap-extend=1000, -nooverlapalign
Multiple Alignment=IR, Tree=UPGMA, Matching against: TRANSFAC
Note: All results files are removed nightly at midnight EST. Please save your results by saving "Webpage, complete".
Download results as a PDF
Click here to run STAMP again.

Multiple Alignment

(Consensus sequence representations shown, but multiple alignment was carried out on the matrices)

```
Motif_1:  -----------------------TCAGAATTCAGTA---------------
Motif_2:  ----------------TTGANATGCAAA-----------------------
Motif_3:  ----------------------GGAAAAGCGCCCCAATTAGTGCCTCCCCC
Motif_4:  -------TCCNGATCCACACGCTACAAGGCA--------------------
Motif_5:  -------------------CAATMCMNAGNACCAG----------------
Motif_6:  ---------ACCNYCCKNNKKTNSCAAGCYGCGG-----------------
Motif_7:  GCGAACCTCCGGAAGTGACAGTAACAGAGA---------------------
Motif_8:  ------CAATAAYATGGATSTACACARCACATG------------------
Motif_9:  ---------------------CAACAAAGANCGCGCCCNCNTTTTC-----
Motif_10: -------------------AAWTGCAAAT----------------------
Motif_11: ---------------CCCWCCNCCCANCYCC--------------------
Motif_12: ------------CACAYACRCANACACAMN---------------------
```

Familial Profile:
(click for matrix)

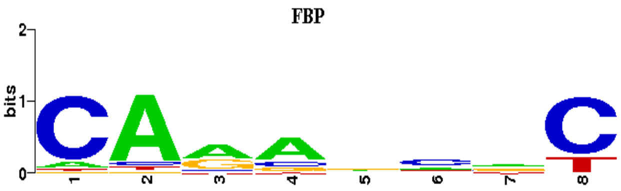

Motif Tree

Tree (drawn by Phylip)
Click here for Newick-format tree (viewable with MEGA)

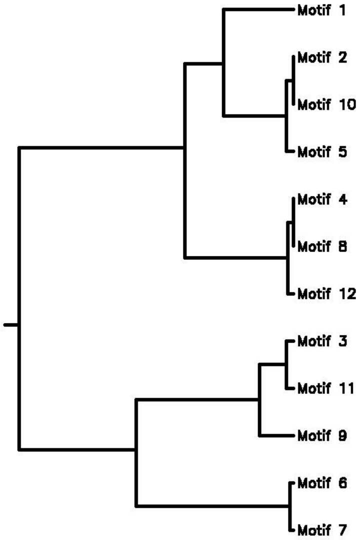

Input Motif	Best match in TRANSFAC
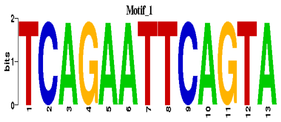 Motif_1	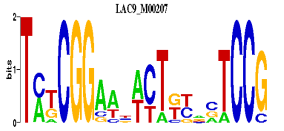 LAC9_M00207 (E val: 8.9124e-06)
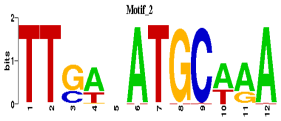 Motif_2	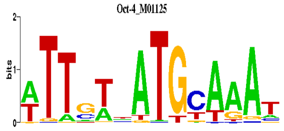 Oct-4_M01125 (E val: 4.4520e-14)
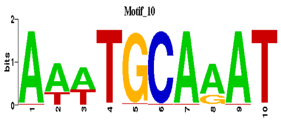 Motif_10	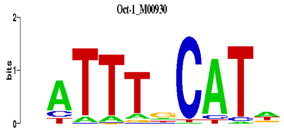 Oct-1_M00930 (E val: 1.0206e-11)
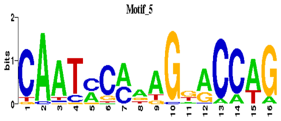 Motif_5	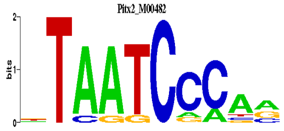 Pitx2_M00482 (E val: 7.7511e-05)
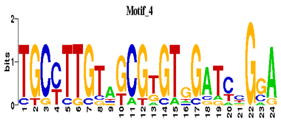 Motif_4	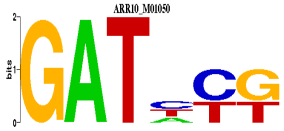 ARR10_M01050 (E val: 4.4376e-05)
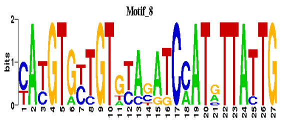 Motif_8	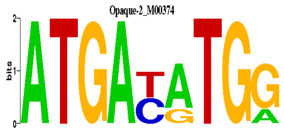 Opaque-2_M00374 (E val: 3.9476e-06)
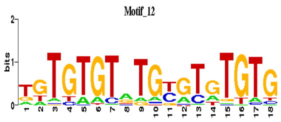 Motif_12	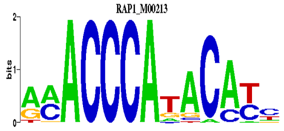 RAP1_M00213 (E val: 5.1544e-07)
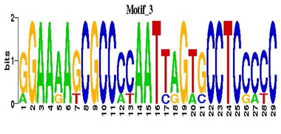 Motif_3	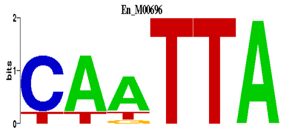 En_M00696

	(E val: 2.3193e-06)
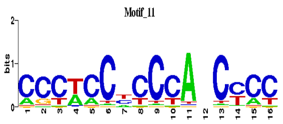 Motif_11	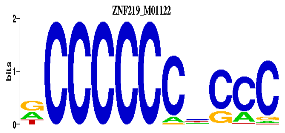 ZNF219_M01122 (E val: 5.1682e-07)
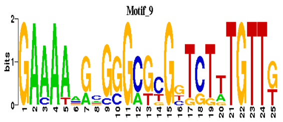 Motif_9	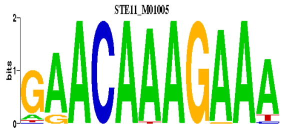 STE11_M01005 (E val: 1.2779e-05)
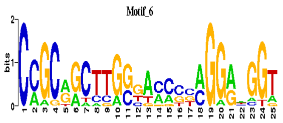 Motif_6	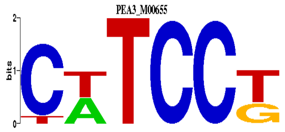 PEA3_M00655 (E val: 1.3863e-05)
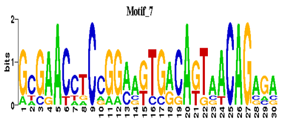 Motif_7	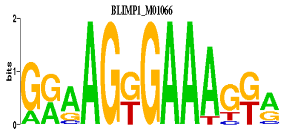 BLIMP1_M01066 (E val: 8.8016e-09)

Motif Similarity Matches

Motif_1

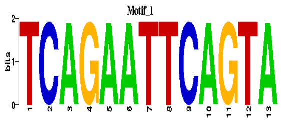

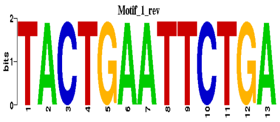

forward *reverse compliment*

Name	*E value*	*Alignment*	*Motif*
LAC9_M00207	8.9124e-06	`-------TACTGAATTCTGA--`<br>`NCGGANNRMAGWNWTCCGMKA`	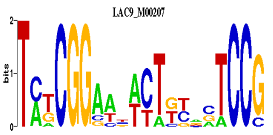
PXR_M01153	2.8134e-04	`TCAGAATTCAGTA`<br>`---NAGTTCA---`	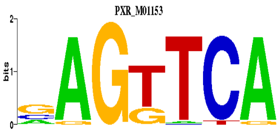
HNF4alpha_M00638	3.0006e-04	`--TCAGAATTCAGTA`<br>`NKKCAAAGKTCA---`	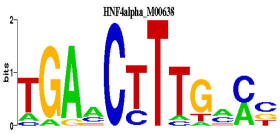

Pax-5_M00144	3.1125e-04	`---------------TCAGAATTCAGTA`<br>`NNNRYNGTYMCGCTYNANNNNNCNNNY`	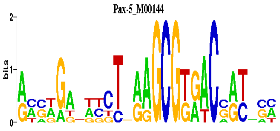
WRKY_M00681	4.2784e-04	`TCAGAATTCAGTA-`<br>`----TAYTYRGTCA`	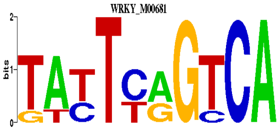
STAT5A_M00460	1.1024e-03	`--TCAGAATTCAGTA--------`<br>`TCNNNGAANNNNNNTTCCNNGNR`	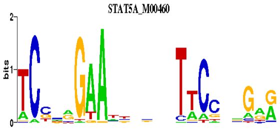
CF1A_M01098	1.9102e-03	`-TCAGAATTCAGTA-`<br>`NTTTNAAYTNATTAW`	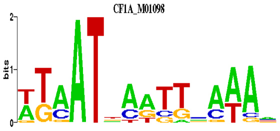
HNF6_M00639	2.3155e-03	`-TACTGAATTCTGA`<br>`NTATTGATTTN---`	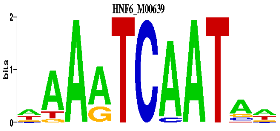
POU6F1_M00465	2.7103e-03	`TACTGAATTCTGA`<br>`--ATAAWTTATG-`	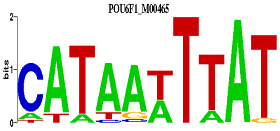
AP-3_M00690	2.9314e-03	`TACTGAATTCTGA`<br>`--CYMAATT----`	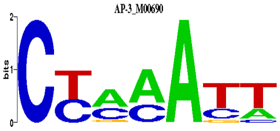

Motif_2

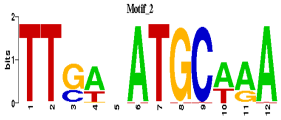

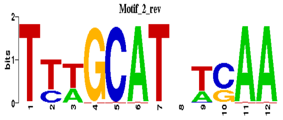

forward *reverse compliment*

Name	*E value*	*Alignment*	*Motif*
Oct-4_M01125	4.4520e-14	`-TTGANATGCAAA-`<br>`WTTSTNATGCAAAT`	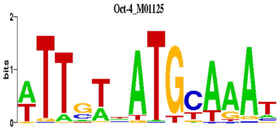
Oct-4_M01124	6.0190e-11	`--TTTGCATNTCAA`<br>`NWTTWGCAWNACAA`	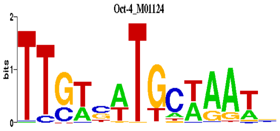

Oct-1_M00342	4.4049e-06	--TTTGCATNTCAA<br>NATTTGCAT-----	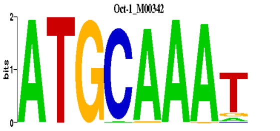
Oct-1_M00930	5.1738e-06	TTGANATGCAAA--<br>----NATGNAAATN	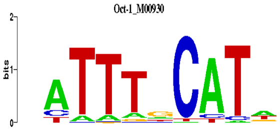
Oct-1_M00161	9.9342e-06	---TTTGCATNTCAA<br>KNATTTGCATAYY--	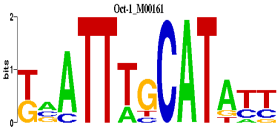
Octamer_M00795	2.1211e-05	--TTTGCATNTCAA<br>NATTTGCATN----	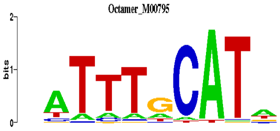
Oct-1_M00135	4.9845e-05	------TTTGCATNTCAA<br>NNNANATTTGCATANNNN	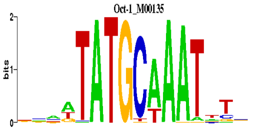
Sry-beta_M00666	7.1234e-05	TTGANATGCAAA<br>-AGWGATGC---	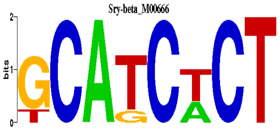
MADS-B_M00404	1.1431e-04	TTTGCATNTCAA--<br>TTTSCATTTTTRGN	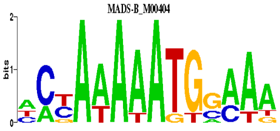
MADS-A_M00408	1.1473e-04	TTTGCATNTCAA---<br>TTTCCATTTTTNNWN	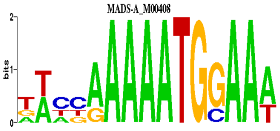

Motif_10

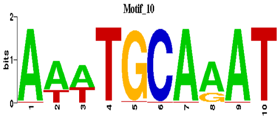

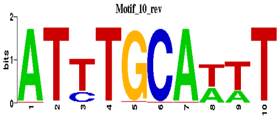

forward *reverse compliment*

Name	*E value*	*Alignment*	*Motif*
Oct-1_M00930	1.0206e-11	AAWTGCAAAT-<br>-NATGNAAATN	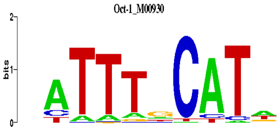
Oct-1_M00161	4.5951e-11	-AAWTGCAAAT--<br>RRTATGCAAATNM	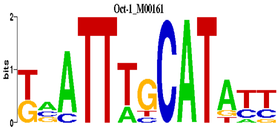

Oct-1_M00342	2.9351e-10	AAWTGCAAAT-<br>--ATGCAAATN	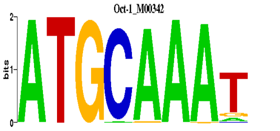
Oct-4_M01125	1.2209e-09	ATTTGCAWTT----<br>ATTTGCATNASAAW	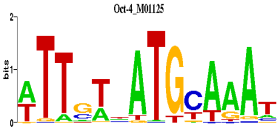
Oct-1_M00135	2.0239e-09	---AAWTGCAAAT-----<br>NNNNTATGCAAATNTNNN	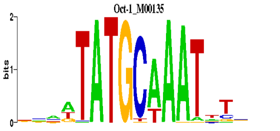
Octamer_M00795	3.7860e-09	AAWTGCAAAT-<br>-NATGCAAATN	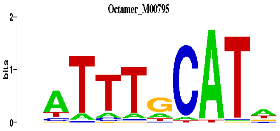
OCT-x_M00210	3.9220e-08	AAWTGCAAAT--<br>RTATGCAAATNA	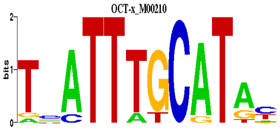
Oct-1_M00195	2.5267e-07	---ATTTGCAWTT-<br>NTNATTTGCATNNN	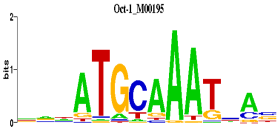
MADS-B_M00404	6.1512e-07	ATTTGCAWTT-----<br>-TTTSCATTTTTRGN	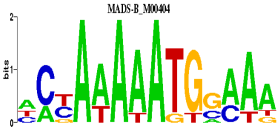
MADS-A_M00408	1.4312e-06	------AAWTGCAAAT<br>NWNNAAAAATGGAAA-	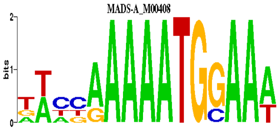

Motif_5

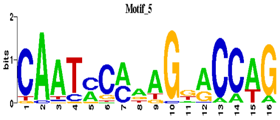 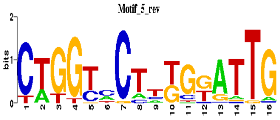

forward *reverse compliment*

Name	*E value*	*Alignment*	*Motif*
Pitx2_M00482	7.7511e-05	CTGGTNCTNKGKATTG-<br>-------NTGGGATTAN	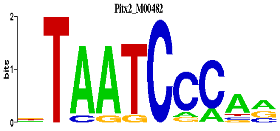

LUN-1_M00480 1.7159e-04

```
CAATMCMNAGNACCAG--
--TCCCAAAGTAGCTGGG
```

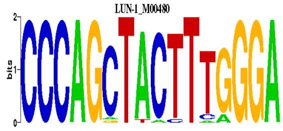

MRF-2_M00454 2.6489e-04

```
CTGGTNCTNKGKATTG----
--------KNNGTATTGTNSW
```

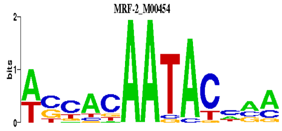

LEF1_M00805 5.3104e-04

```
CTGGTNCTNKGKATTG
------CTTTG-----
```

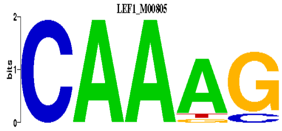

PPARalpha-RXRalpha_M00518 8.5130e-04

```
--CAATMCMNAGNACCAG
CNTSACCCCNATGACCNN
```

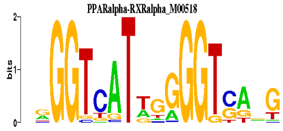

EBF_M00977 1.1559e-03

```
CAATMCMNAGNACCAG
---NCYCWRGGGA---
```

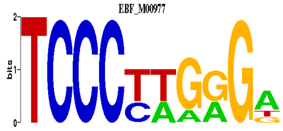

HOXA7_M01108 1.9510e-03

```
CTGGTNCTNKGKATTG
----------AGATTG
```

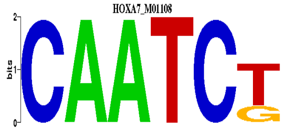

PBF_M01130 2.0304e-03

```
CAATMCMNAGNACCAG
-------AAAGG-----
```

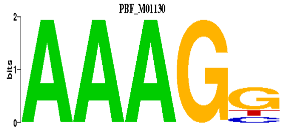

AR_M00953 3.2172e-03

```
-------CAATMCMNAGNACCAG---
NNNNNNAGAACACNNTGTNCYNNNNN
```

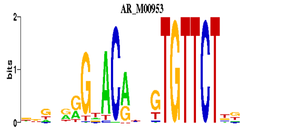

OCT-x_M00210 4.7987e-03

```
CTGGTNCTNKGKATTG
----TNATTTGCATAY
```

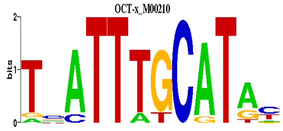

Motif_4

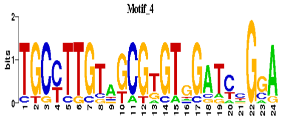

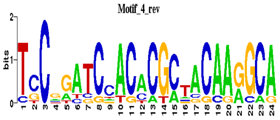

forward *reverse compliment*

Name *E value* *Alignment* *Motif*

ARR10_M01050	4.4376e-05	TCCNGATCCACACGCTACAAGGCA<br>--MRNATC----------------	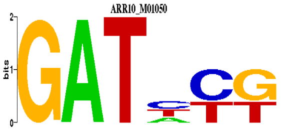
FACB_M00389	1.0170e-04	---TCCNGATCCACACGCTACAAGGCA<br>ANNNGCNRNGMNAAAMNCTGCNNNN--	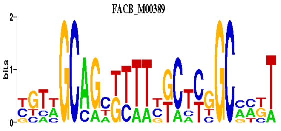
Pax-5_M00144	2.8090e-03	-----TGCCTTGTAGCGTGTGGATCNGGA<br>RNNNGNNNNNTNRAGCGKRACNRYNNN--	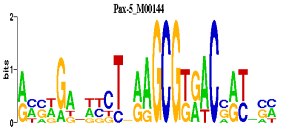
Tra-1_M01049	4.1863e-03	TGCCTTGTAGCGTGTGGATCNGGA<br>-----------GGGNGGT------	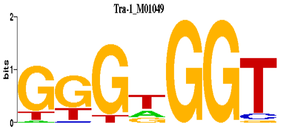
Pax-4_M00373	5.4883e-03	TGCCTTGTAGCGTGTGGATCNGGA<br>-GNNKTCANGCGTGNNSNNNN---	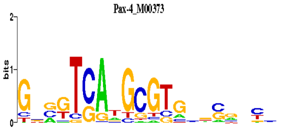
Sp3_M00665	6.7747e-03	TCCNGATCCACACGCTACAAGGCA<br>----------CCCYSCCCAAGKS-	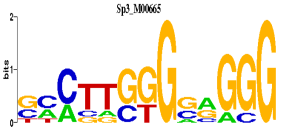
LEF1_M00805	7.4009e-03	TGCCTTGTAGCGTGTGGATCNGGA<br>--CTTTG-----------------	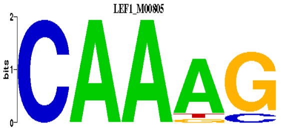
HAC1_M00730	7.5088e-03	TGCCTTGTAGCGTGTGGATCNGGA<br>-----GMCAGCGTGTC--------	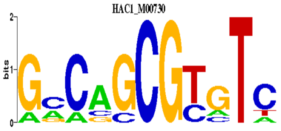
NF-kappaB_M00051	9.5336e-03	TGCCTTGTAGCGTGTGGATCNGGA<br>-------------GGGRATCCC--	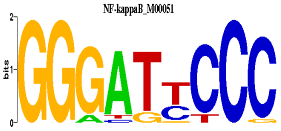
CACD_M01113	1.1315e-02	TGCCTTGTAGCGTGTGGATCNGGA<br>---------GGGYGTG--------	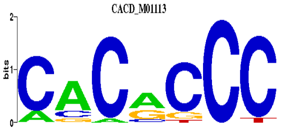

Motif_8

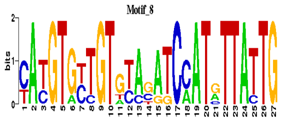

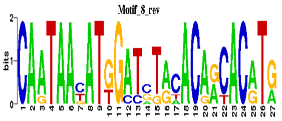

forward *reverse compliment*

Name	*E value*	*Alignment*	*Motif*
Opaque-2_M00374	3.9476e-06	CAATAAYATGGATSTACACARCACATG<br>--ATGAYATGG----------------	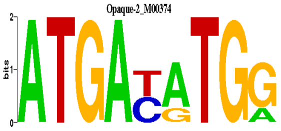 Opaque-2_M00374
TWI_M01103	1.3335e-05	CAATAAYATGGATSTACACARCACATG<br>-------------NNGCNNMACAYAT-	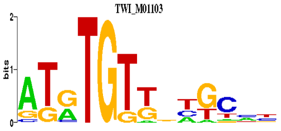 TWI_M01103
CRP_M00202	4.3897e-05	CATGTGYTGTGTASATCCATRTTATTG<br>-WANTGTGANNYNNNTCACAWTWATT-	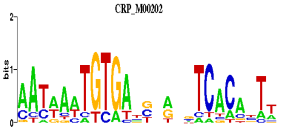 CRP_M00202
TFE_M01029	1.4757e-04	CAATAAYATGGATSTACACARCACATG<br>--------------------NCACATG	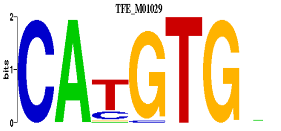 TFE_M01029
ATHB-5_M00503	3.1301e-04	CAATAAYATGGATSTACACARCACATG<br>CAATNATT-------------------	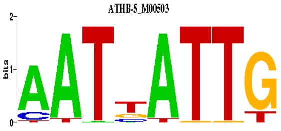 ATHB-5_M00503
bHLH66_M01054	5.8229e-04	CAATAAYATGGATSTACACARCACATG<br>--------------------GCACGTG	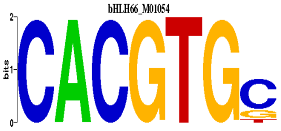 bHLH66_M01054
BRCA1-USF2_M01082	9.7729e-04	CAATAAYATGGATSTACACARCACATG<br>------------------CAACNNN--	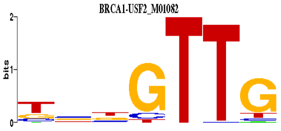 BRCA1-USF2_M01082
MATalpha2_M00031	1.6817e-03	CAATAAYATGGATSTACACARCACATG<br>-NWNNACAYG-----------------	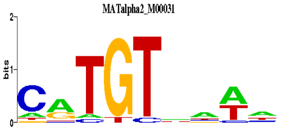 MATalpha2_M00031
RITA-1_M00660	1.8787e-03	CAATAAYATGGATSTACACARCACATG<br>---------------------SACGTR	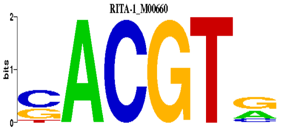 RITA-1_M00660
POU3F2_M00464	2.6381e-03	CAATAAYATGGATSTACACARCACATG<br>-ATTARCATA-----------------	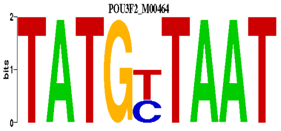 POU3F2_M00464

Motif_12

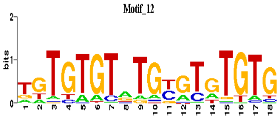

forward

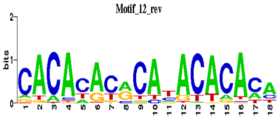

reverse compliment

Name	*E value*	*Alignment*	*Motif*
RAP1_M00213	5.1544e-07	NKTGTGTNTGYGTRTGTG<br>--NRTGTATGGGTKY---	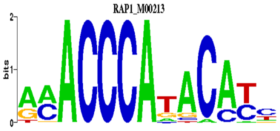
Tax-CREB_M00115	4.3219e-05	NKTGTGTNTGYGTRTGTG<br>-GGGGRTATGCGTCA---	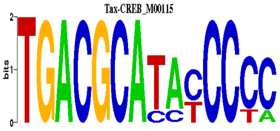
FOXP1_M00987	6.0635e-05	-CACAYACRCANACACAMN<br>ATAAAAAACAACACAAATA	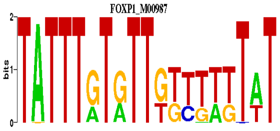
NGFI-C_M00244	6.8421e-05	-CACAYACRCANACACAMN<br>CCRCCCACGCA--------	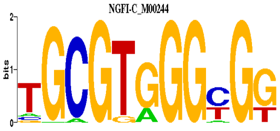
Egr-3_M00245	1.5210e-04	CACAYACRCANACACAMN<br>-----ACGCCCACGCA--	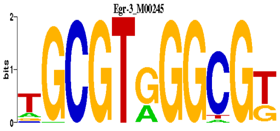
Egr-2_M00246	2.0592e-04	CACAYACRCANACACAMN<br>-----MCGCCCACGCA--	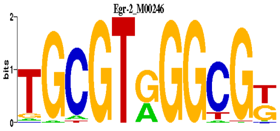
Pit-1_M00802	2.0641e-04	CACAYACRCANACACAMN<br>-MTTCATNNWTATNNNNN	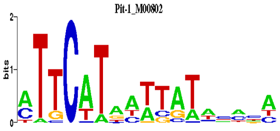
Egr-1_M00243	3.5130e-04	NKTGTGTNTGYGTRTGTG<br>--TGCGTGGGCGK-----	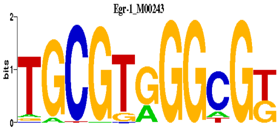
CACD_M01113	6.4645e-04	CACAYACRCANACACAMN<br>--CACRCCC---------	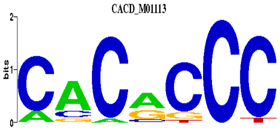
HFH4_M00742	2.2067e-03	NKTGTGTNTGYGTRTGTG<br>--WKTGTTTGTTTA----	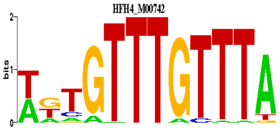

Motif_3

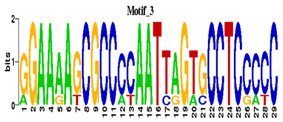

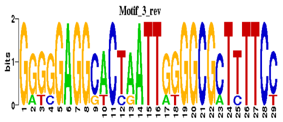

forward *reverse compliment*

Name	*E value*	*Alignment*	*Motif*
En_M00696	2.3193e-06	GGAAAAGCGCCCCAATTAGTGCCTCCCCC<br>------------CAATTA-----------	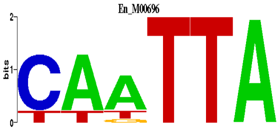
HOXA5_M00023	2.8121e-06	--GGGGGAGGCACTAATTGGGGCGCTTTTCC<br>NGRNNNNNNNNANTAATGRNGNNNNNNGC--	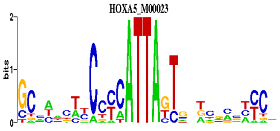
HOXA4_M00640	4.2207e-06	GGAAAAGCGCCCCAATTAGTGCCTCCCCC<br>-----------CYAATTW-----------	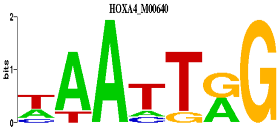
TFII-I_M00706	5.4830e-06	GGAAAAGCGCCCCAATTAGTGCCTCCCCC<br>---------------------CCTMCNTC	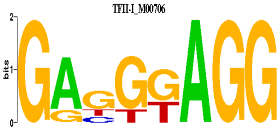
Pax-4_M00380	1.5795e-05	-GGGGGAGGCACTAATTGGGGCGCTTTTCC<br>NGNGTGNNNNNNNNNNNNNNNNTAWTTTN-	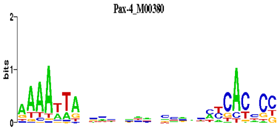
ATHB-5_M00503	4.2772e-05	GGAAAAGCGCCCCAATTAGTGCCTCCCCC<br>-------------AATNATTG--------	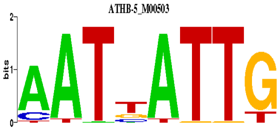
UF1H3BETA_M01068	7.4050e-05	--GGGGGAGGCACTAATTGGGGCGCTTTTCC<br>GYGGGGGAGGGGC------------------	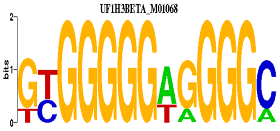
MAZ_M00649	1.0199e-04	GGAAAAGCGCCCCAATTAGTGCCTCCCCC<br>--------------------CCCTCCC--	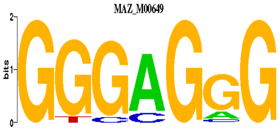
Nkx2-5_M00241	1.2150e-04	GGAAAAGCGCCCCAATTAGTGCCTCCCCC<br>------------NAATTAW----------	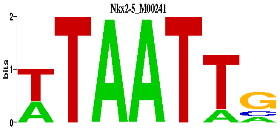
E2F-1_M00939	4.6967e-04	GGAAAAGCGCCCCAATTAGTGCCTCCCCC<br>-------CGCGCSAA--------------	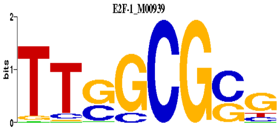

Motif_11

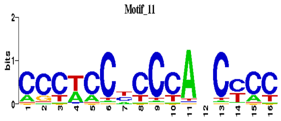

forward

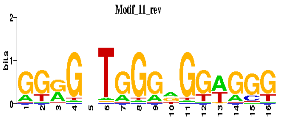

reverse compliment

Name	*E value*	*Alignment*	*Motif*
ZNF219_M01122	5.1682e-07	-CCCWCCNCCCANCYCC<br>NCCCCCCNCCC------	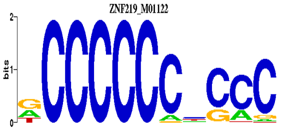
VDR_M00444	1.4762e-06	CCCWCCNCCCANCYCC<br>-TSWCCYYNYTNMCC-	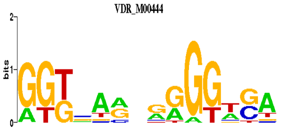
UF1H3BETA_M01068	3.3541e-06	--CCCWCCNCCCANCYCC<br>GCCCCTCCCCCRC-----	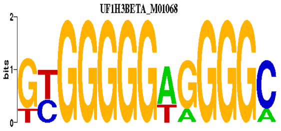
KROX_M00982	1.2985e-05	GGRGNTGGGNGGWGGG<br>GNNGNGGGGGCGG---	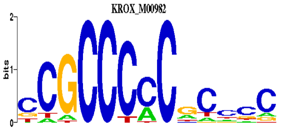
MAZ_M00649	1.6038e-05	CCCWCCNCCCANCYCC<br>CCCTCCC---------	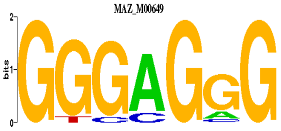
AtMYB-84_M00970	4.0469e-05	CCCWCCNCCCANCYCC<br>----SCACCTACCNCC	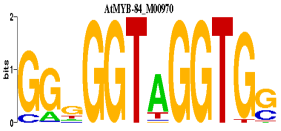
CAC-binding_M00720	7.2713e-05	GGRGNTGGGNGGWGGG<br>---------RGGSTGGG	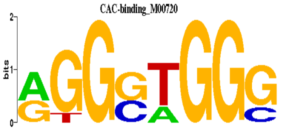
Lyf-1_M00141	1.6046e-04	CCCWCCNCCCANCYCC<br>----YCTCCCAA----	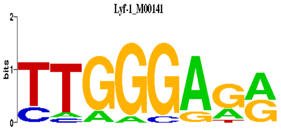
Tra-1_M01048	2.1934e-04	GGRGNTGGGNGGWGGG<br>------GGGWGGT---	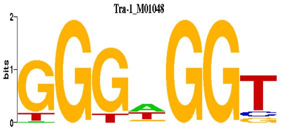
TFII-I_M00706	2.3594e-04	CCCWCCNCCCANCYCC<br>-CCTMCNTC-------	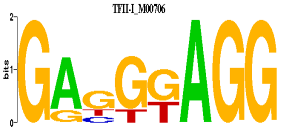

Motif_9

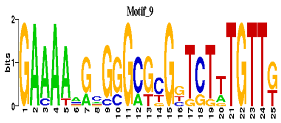

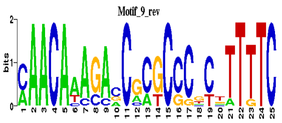

forward *reverse compliment*

Name	*E value*	*Alignment*	*Motif*
STE11_M01005	1.2779e-05	CAACAAAGANCGCGCCCNCNTTTTC<br>GAACAAAGAAA---------------	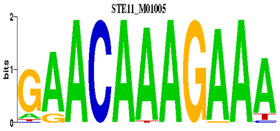
Mat1-Mc_M00275	3.3878e-05	----CAACAAAGANCGCGCCCNCNTTTTC<br>NRMNNAACAAAGANRYC------------	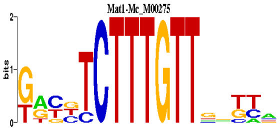
SRY_M00148	4.0988e-05	CAACAAAGANCGCGCCCNCNTTTTC<br>-AACANM------------------	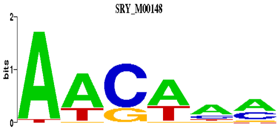
ZAP1_M00735	8.1564e-05	CAACAAAGANCGCGCCCNCNTTTTC<br>------TGACCGAGY----------	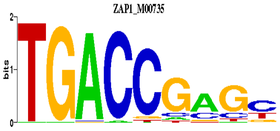
SOX_M01014	1.1660e-04	---CAACAAAGANCGCGCCCNCNTTTTC<br>NCNTAACAAAGA----------------	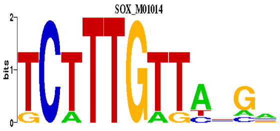
ZF5_M00333	1.8894e-04	CAACAAAGANCGCGCCCNCNTTTTC<br>-------NWGCGCGYNYNY------	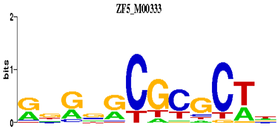
LEF1_M00805	1.9076e-04	GAAAANGNGGGCGCGNTCTTTGTTG<br>-----------------CTTTG---	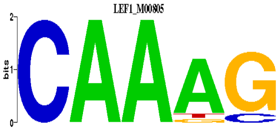
NRSF_M00256	2.1431e-04	----CAACAAAGANCGCGCCCNCNTTTTC<br>TCAGCACCACGGACAGCRSC---------	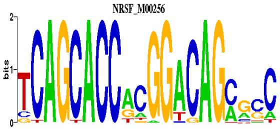
HFH4_M00742	3.2897e-04	-CAACAAAGANCGCGCCCNCNTTTTC<br>TAAACAAACAMW--------------	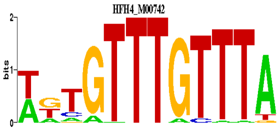
KROX_M00982	6.9677e-04	GAAAANGNGGGCGCGNTCTTTGTTG<br>---GNNGNGGGGGCGG---------	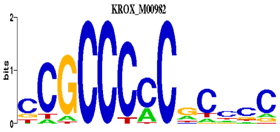

Motif_6

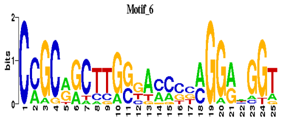

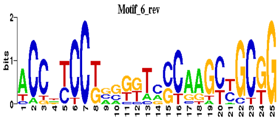

forward *reverse compliment*

Name	*E value*	*Alignment*	*Motif*
PEA3_M00655	1.3863e-05	CCGCRGCTTGSNAMMNNNMGGRNGGT<br>-----------------MGGAWG--	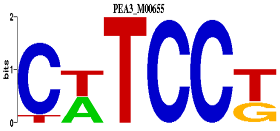
Tra-1_M01048	5.2093e-05	ACCNYCCKNNKKTNSCAAGCYGCGG<br>ACCWCCC------------------	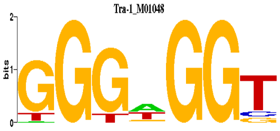
PU.1_M00658	3.8276e-04	ACCNYCCKNNKKTNSCAAGCYGCGG<br>--CTTCCTC----------------	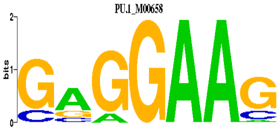
LBP-1_M00644	3.8363e-04	CCGCRGCTTGSNAMMNNNMGGRNGGT<br>--SCAGCN------------------	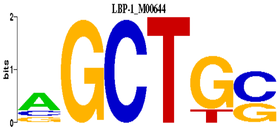
c-Ets-1_M00743	3.9782e-04	ACCNYCCKNNKKTNSCAAGCYGCGG<br>-GCTTCCK-----------------	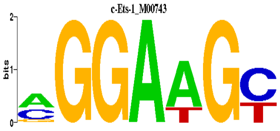
Tra-1_M01049	9.7222e-04	ACCNYCCKNNKKTNSCAAGCYGCGG<br>ACCNCCC------------------	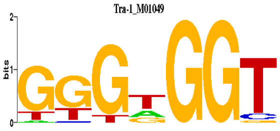
Ets_M00971	1.1419e-03	CCGCRGCTTGSNAMMNNNMGGRNGGT<br>----------------NAGGAAG--	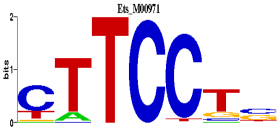
Olf-1_M00261	3.1243e-03	---ACCNYCCKNNKKTNSCAAGCYGCGG<br>NCMNNYTCCCNRGGGANTNGN-------	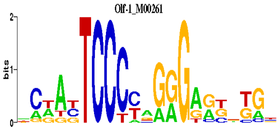
Tel-2_M00678	3.3696e-03	CCGCRGCTTGSNAMMNNNMGGRNGGT<br>----------------CAGGAAGTA	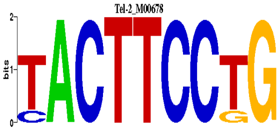
c-Ets-1_p54_M00032	3.5880e-03	ACCNYCCKNNKKTNSCAAGCYGCGG<br>NRCWTCCKG----------------	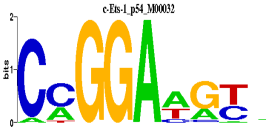

Motif_7

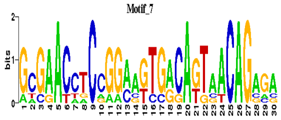
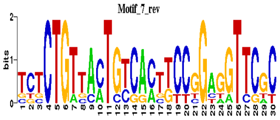

forward *reverse compliment*

Name	*E value*	*Alignment*	*Motif*
BLIMP1_M01066	8.8016e-09	TCTCTGTTACTGTCACTTCCGGAGGTTCGC<br>--------YMCTTTCMCTTYC----------	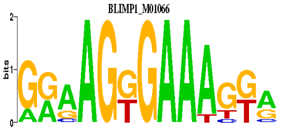
GABP_M00341	1.3725e-07	TCTCTGTTACTGTCACTTCCGGAGGTTCGC<br>------------YGCNCTTCCGG--------	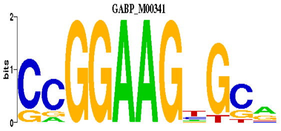
Nrf-2_M00108	4.2402e-07	GCGAACCTCCGGAAGTGACAGTAACAGAGA<br>---------CCGGAAGNS-------------	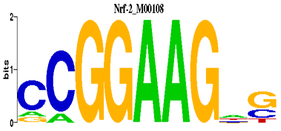
c-Ets-1_p54_M00032	5.7446e-05	GCGAACCTCCGGAAGTGACAGTAACAGAGA<br>---------CMGGAWGYN-------------	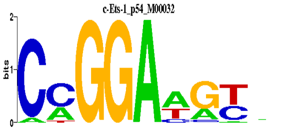
Tel-2_M00678	1.3932e-04	TCTCTGTTACTGTCACTTCCGGAGGTTCGC<br>--------------TACTTCCTG--------	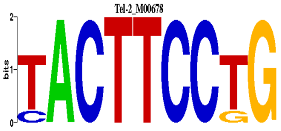
ANT_M00501	1.4112e-04	GCGAACCTCCGGAAGTGACAGTAACAGAGA<br>-----CMTYGGGAWNTGT------------	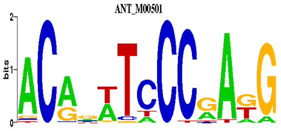
SOX_M01014	1.4978e-04	GCGAACCTCCGGAAGTGACAGTAACAGAGA<br>------------------NCNTAACAAAGA	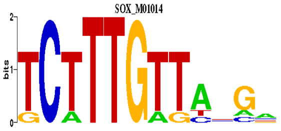
LXR_M00766	2.4248e-04	TCTCTGTTACTGTCACTTCCGGAGGTTCGC<br>----------GGGTTACTNNCGGTC------	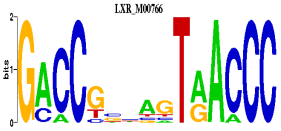
HSF_M00641	4.1429e-04	TCTCTGTTACTGTCACTTCCGGAGGTTCGC<br>------------------TCNMGNRGYTTC-	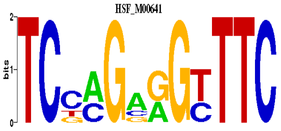
STAT1_M00492	4.2149e-04	GCGAACCTCCGGAAGTGACAGTAACAGAGA<br>----------SGGAANT--------------	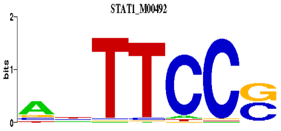

Sequence logo generation powered by weblogo
STAMP is written by Shaun Mahony
