# Supplementary material for: In silico identification of a core regulatory network of OCT4 in human embryonic stem cells using an integrated approach
Source: BMC Genomics. 2009 Jul 15;10:314. doi: 10.1186/1471-2164-10-314 (PMC2714862; doi:10.1186/1471-2164-10-314)
Supplement: Additional file 9 — Motif database matching results- JASPAR. The pdf file contains the results from comparing the 12 discovered motifs to the JASPAR database (v3, [31]) using the STAMP tool [32]. [file 1471-2164-10-314-S9.pdf]

Jump to: Multiple Alignment Motif Tree Motif Matching
Input file: 12 motifs loaded
Settings: Metric=PCC, Alignment=SWU, Gap-open=1000, Gap-extend=1000, -nooverlapalign
Multiple Alignment=IR, Tree=UPGMA, Matching against: JASPAR
Note: All results files are removed nightly at midnight EST. Please save your results by saving "Webpage, complete".
Download results as a PDF
Click here to run STAMP again.

Multiple Alignment

(Consensus sequence representations shown, but multiple alignment was carried out on the matrices)

```
Motif_1:  -----------------------TCAGAATTCAGTA---------------
Motif_2:  ----------------TTGANATGCAAA-----------------------
Motif_3:  ----------------------GGAAAAGCGCCCCAATTAGTGCCTCCCCC
Motif_4:  -------TCCNGATCCACACGCTACAAGGCA--------------------
Motif_5:  -------------------CAATMCMNAGNACCAG----------------
Motif_6:  ---------ACCNYCCKNNKKTNSCAAGCYGCGG-----------------
Motif_7:  GCGAACCTCCGGAAGTGACAGTAACAGAGA---------------------
Motif_8:  ------CAATAAYATGGATSTACACARCACATG------------------
Motif_9:  ---------------------CAACAAAGANCGCGCCCNCNTTTTC-----
Motif_10: -------------------AAWTGCAAAT----------------------
Motif_11: ---------------CCCWCCNCCCANCYCC--------------------
Motif_12: ------------CACAYACRCANACACAMN---------------------
```

Familial Profile:
(click for matrix)

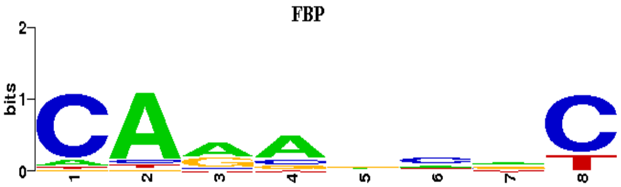

Motif Tree

Tree (drawn by Phylip)
Click here for Newick-format tree (viewable with MEGA)

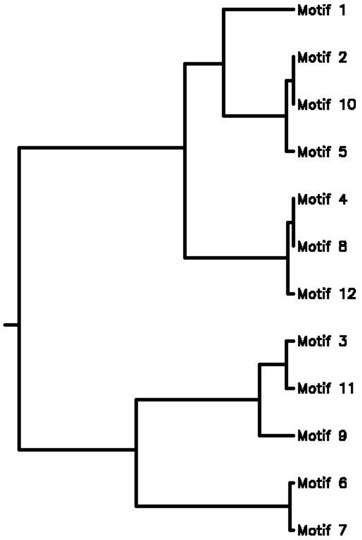

Input Motif	Best match in JASPAR
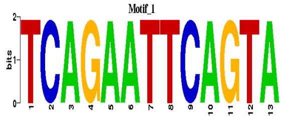 Motif_1	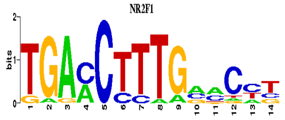 NR2F1 (E val: 2.5413e-03)
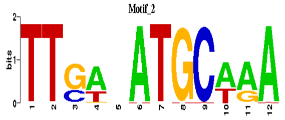 Motif_2	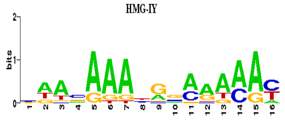 HMG-IY (E val: 1.7118e-05)
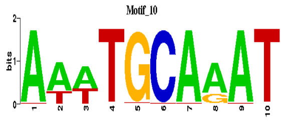 Motif_10	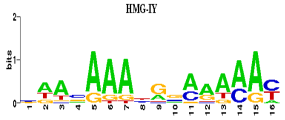 HMG-IY (E val: 3.3314e-06)
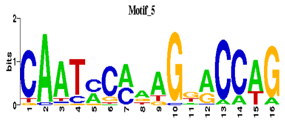 Motif_5	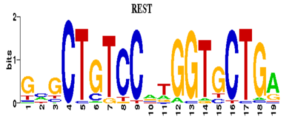 REST (E val: 9.2402e-03)
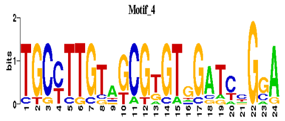 Motif_4	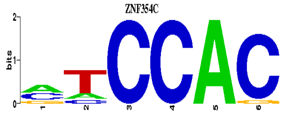 ZNF354C (E val: 3.9705e-05)
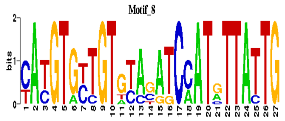 Motif_8	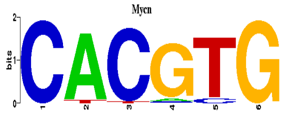 Mycn (E val: 7.5450e-04)
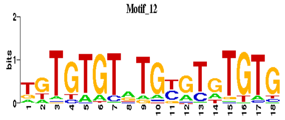 Motif_12	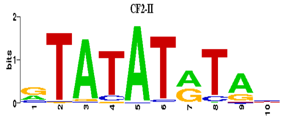 CF2-II (E val: 5.0163e-03)
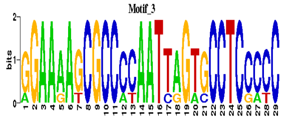 Motif_3	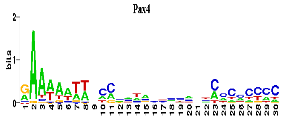 Pax4

	(E val: 4.8136e-11)
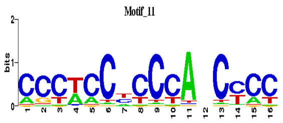<br>Motif_11	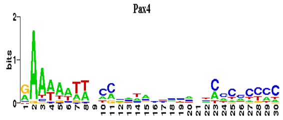<br>Pax4<br>(E val: 6.5278e-04)
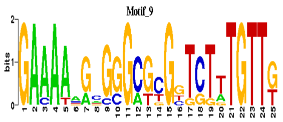<br>Motif_9	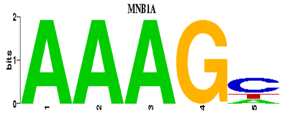<br>MNB1A<br>(E val: 3.1854e-04)
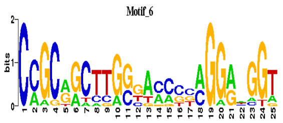<br>Motif_6	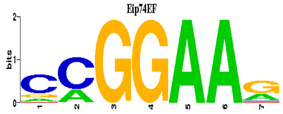<br>Eip74EF<br>(E val: 3.0567e-04)
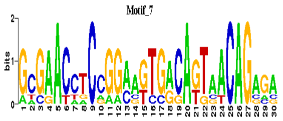<br>Motif_7	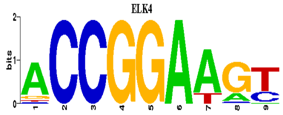<br>ELK4<br>(E val: 2.2790e-06)

Motif Similarity Matches

Motif_1

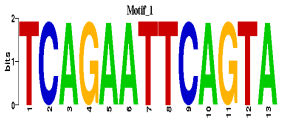
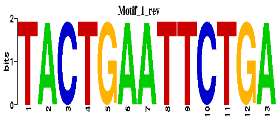

forward *reverse compliment*

Name	*E value*	*Alignment*	*Motif*
NR2F1	2.5413e-03	`----TCAGAATTCAGTA`<br>`NNGKNCAAAGKTCA---`	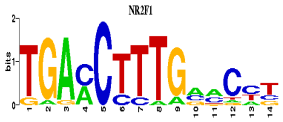
Lhx3_v2HOMEO	7.9667e-03	`TCAGAATTCAGTA---`<br>`---WAATTAATTAWYN`	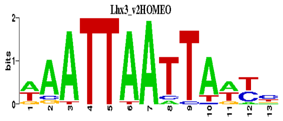
Prrx2	8.5980e-03	`TCAGAATTCAGTA`<br>`---TAATT-----`	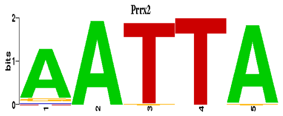

NKX3-1	9.7950e-03	TCAGAATTCAGTA-<br>-------TAAGTAT	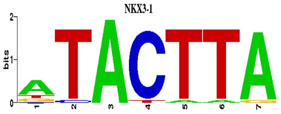
Pax6	1.2029e-02	TACTGAATTCTGA-<br>MANTSAWGCGTGAA	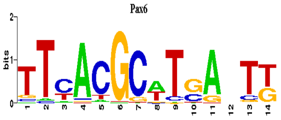
Staf	1.2673e-02	-TACTGAATTCTGA------<br>KCNNKGMANNMTGGGANNNN	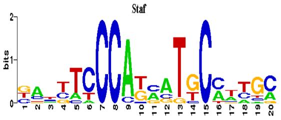
Lhx3	1.5097e-02	-TACTGAATTCTGA<br>WTAATTART-----	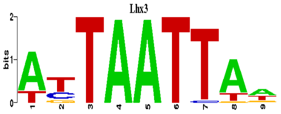
HNF4A	2.0847e-02	TACTGAATTCTGA---<br>---TGNMCTTTGNNCY	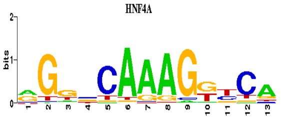
NF-kappaB	3.2152e-02	TACTGAATTCTGA<br>--GGGAMTTYCC-	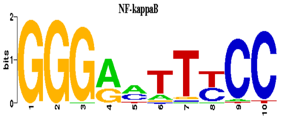
Athb-1	3.3186e-02	TCAGAATTCAGTA<br>-AATAATTN----	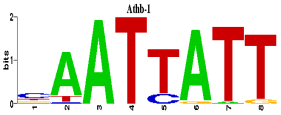

Motif_2

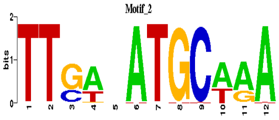

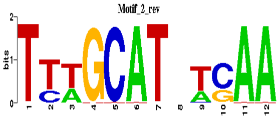

forward *reverse compliment*

Name	*E value*	*Alignment*	*Motif*
HMG-IY	1.7118e-05	---TTTGCATNTCAA-<br>RTTTYKNYNTTTNTNN	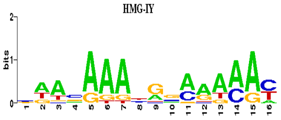
SQUA	1.0237e-03	-TTGANATGCAAA-<br>MCAWAWATRGNAAN	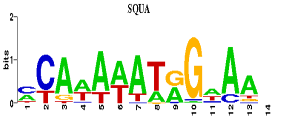

Broad-complex_2	3.4060e-02	TTGANATGCAAA<br>---AAATAGNW-	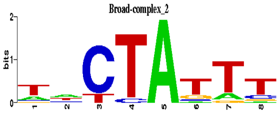
NR2F1	3.4076e-02	-----TTTGCATNTCAA<br>TGAMCTTTGNMCNN---	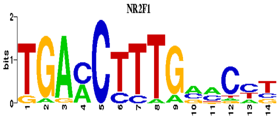
HMG-1	3.9254e-02	TTTGCATNTCAA-<br>----GANNACAAC	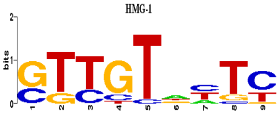
STAT1	3.9268e-02	TTGANATGCAAA--<br>RGAAANNGAAACTN	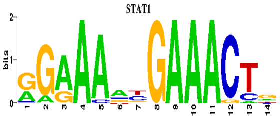
RELA	4.2426e-02	TTTGCATNTCAA<br>-GGGANTTTCC-	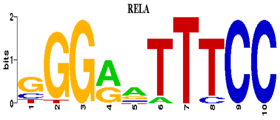
NR1H2-RXRA	7.1076e-02	-TTTGCATNTCAA----<br>STTGACCTTTGACCTTT	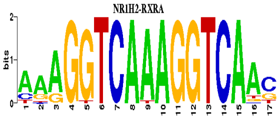
MNB1A	7.9661e-02	TTTGCATNTCAA<br>---NCTTT----	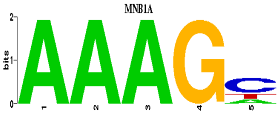
Dl_2	8.2895e-02	TTGANATGCAAA<br>-GGAAAWCCCC-	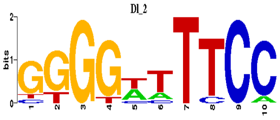

Motif_10

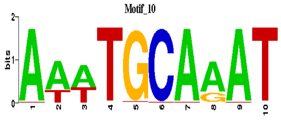

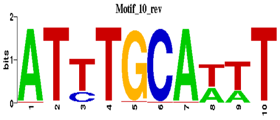

forward *reverse compliment*

Name	*E value*	*Alignment*	*Motif*
HMG-IY	3.3314e-06	--ATTTGCAWTT----<br>RTTTYKNYNTTTNTNN	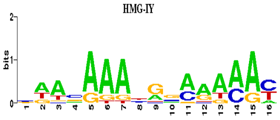
SQUA	9.4765e-05	----AAWTGCAAAT<br>MCAWAWATRGNAAN	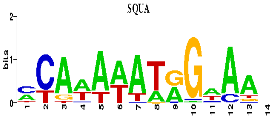

STAT1	8.7206e-04	-ATTTGCAWTT---<br>NAGTTTCNNTTTCY	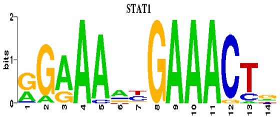
Pax4	1.4392e-03	----AAWTGCAAAT----------------<br>RAAWAWWWNNMNNNNNNNNNNNMNNNNNYMC	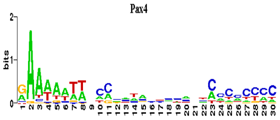
Broad-complex_1	1.5224e-03	-ATTTGCAWTT---<br>NWYTTGTYTATNAN	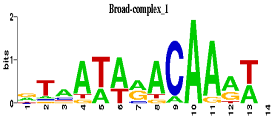
Cebpa	1.4695e-02	ATTTGCAWTT--<br>TTNNNCAAYNNN	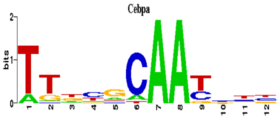
HMG-1	1.7196e-02	AAWTGCAAAT<br>GANNACAAC-	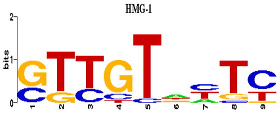
Ddit3-Cebpa	2.2941e-02	--AAWTGCAAAT<br>GSNATTGCAYNN	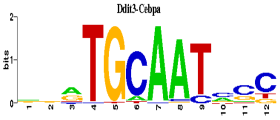
NR2F1	2.4345e-02	----ATTTGCAWTT<br>TGAMCTTTGNMCNN	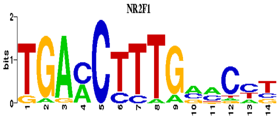
Foxa2	2.9932e-02	AAWTGCAAAT----<br>--AARYAAATANTN	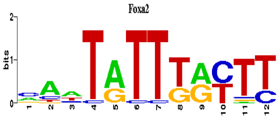

Motif_5

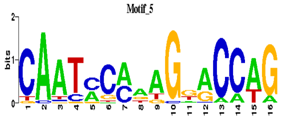

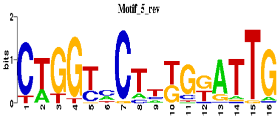

forward *reverse compliment*

Name	*E value*	*Alignment*	*Motif*
REST	9.2402e-03	--CTGGTNCTNKGKATTG-<br>GNGCTGTCCNWGGTGCTGA	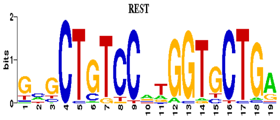

Ddit3-Cebpa	1.3404e-02	CTGGTNCTNKGKATTG-----<br>----------GSNATTGCAYNN	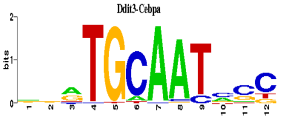
ovo	1.4403e-02	-CTGGTNCTNKGKATTG<br>NCNGTTACW--------	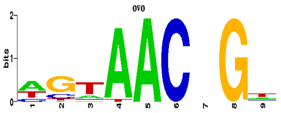
NR1H2-RXRA	3.1690e-02	-CAATMCMNAGNACCAG<br>AAAGGTCAAAGGTCAAS	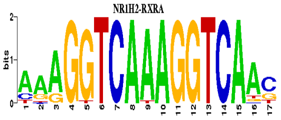
Ar	4.2602e-02	----CAATMCMNAGNACCAG--<br>NNNAGNACANNNTGTNCCNNMN	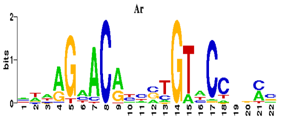
BRCA1	4.3790e-02	CTGGTNCTNKGKATTG-<br>-----------SNGTTGN	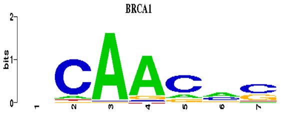
HMG-1	4.6224e-02	CAATMCMNAGNACCAG<br>GANNACAAC-------	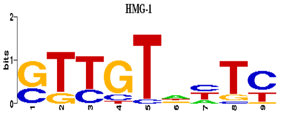
ZNF354C	4.6687e-02	CTGGTNCTNKGKATTG<br>---------GTGGAK--	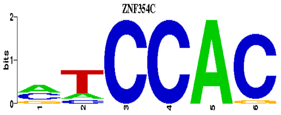
sna	4.7824e-02	CAATMCMNAGNACCAG<br>----------CACCTG	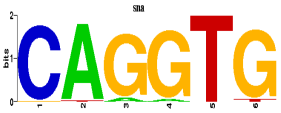
id1	5.0259e-02	CTGGTNCTNKGKATTG<br>TNKYYNNNTNNCG----	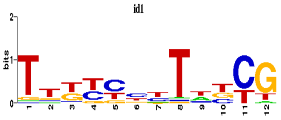

Motif_4

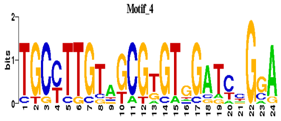

forward

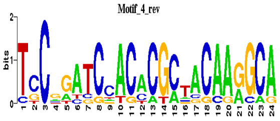

reverse compliment

Name	*E value*	*Alignment*	*Motif*

ZNF354C	3.9705e-05	TGCCTTGTAGCGTGTGGATCNGGA<br>--------------GTGGAK-----	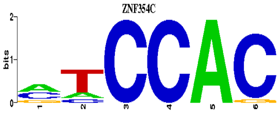
Arnt-Ahr	2.5357e-03	TCCNGATCCACACGCTACAAGGCA<br>-----------CACGCN--------	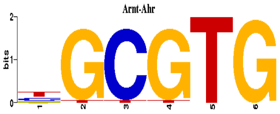
ARR10	6.9143e-03	TCCNGATCCACACGCTACAAGGCA<br>-NMRNATCT---------------	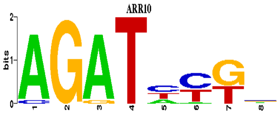
MNB1A	1.2029e-02	TGCCTTGTAGCGTGTGGATCNGGA<br>-NCTTT------------------	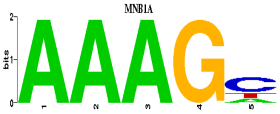
PBF	2.0724e-02	TCCNGATCCACACGCTACAAGGCA<br>------------------AAAGY-	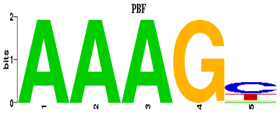
Eip74EF	3.3313e-02	TGCCTTGTAGCGTGTGGATCNGGA<br>----------------YTTCCGG-	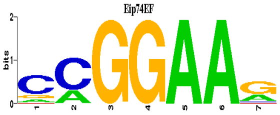
Dof2	4.0010e-02	TGCCTTGTAGCGTGTGGATCNGGA<br>NGCTTT------------------	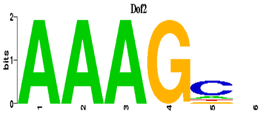
Pax5	5.3635e-02	--TGCCTTGTAGCGTGTGGATCNGGA<br>NGNNNNNYGNWGCGKRRCSR------	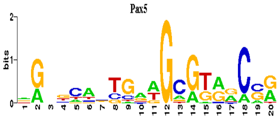
GATA2	5.5833e-02	TGCCTTGTAGCGTGTGGATCNGGA<br>---------------NGATN----	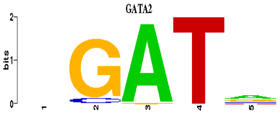
NFKB1	5.6928e-02	TCCNGATCCACACGCTACAAGGCA<br>GGGGAATCCCC-------------	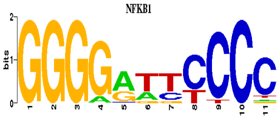

Motif_8

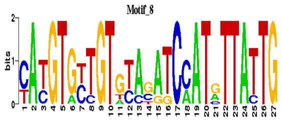

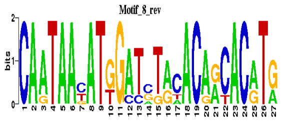

forward *reverse compliment*

Name	*E value*	*Alignment*	*Motif*
Mycn	7.5450e-04	CATGTGYTGTGTASATCCATRTTATTG<br>CACGTG---------------------	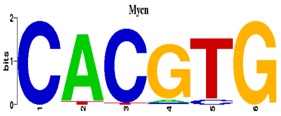
Arnt	1.3445e-03	CAATAAYATGGATSTACACARCACATG<br>---------------------CACGTG	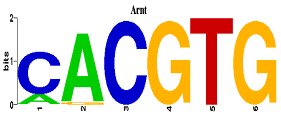
BRCA1	6.4320e-03	CATGTGYTGTGTASATCCATRTTATTG<br>---SNGTTGN-----------------	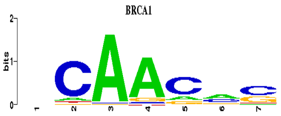
USF1	6.6699e-03	CAATAAYATGGATSTACACARCACATG<br>--------------------NCACGTG	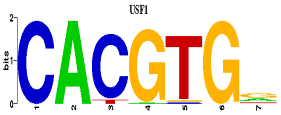
Athb-1	2.0170e-02	CATGTGYTGTGTASATCCATRTTATTG<br>------------------NAATTATT-	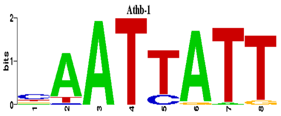
MYC-MAX	2.0730e-02	--CATGTGYTGTGTASATCCATRTTATTG<br>ACCACGTGSTN------------------	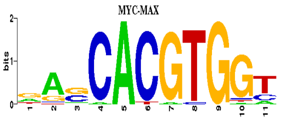
ZNF354C	2.2552e-02	CAATAAYATGGATSTACACARCACATG<br>-------GTGGAK--------------	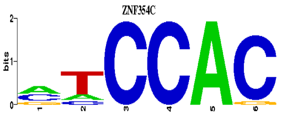
EMBP1	2.3024e-02	-CATGTGYTGTGTASATCCATRTTATTG<br>CCACGTST--------------------	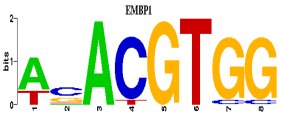
sna	3.7716e-02	CAATAAYATGGATSTACACARCACATG<br>---------------------CACCTG	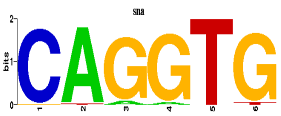
REST	4.1289e-02	CATGTGYTGTGTASATCCATRTTATTG-<br>---------GNGCTGTCCNWGGTGCTGA	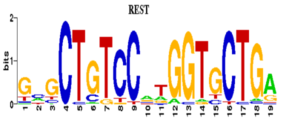

Motif_12

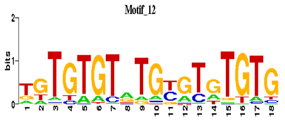

forward

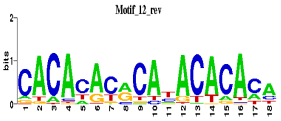

reverse compliment

Name	*E value*	*Alignment*	*Motif*
CF2-II	5.0163e-03	CACAYACRCANACACAMN<br>---NTAYATATAY-----	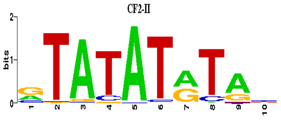
Arnt-Ahr	1.1257e-02	CACAYACRCANACACAMN<br>----CACGCN--------	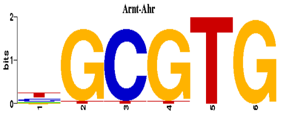
HMG-IY	1.1601e-02	-CACAYACRCANACACAMN<br>NNANAAANRNMRAAAY---	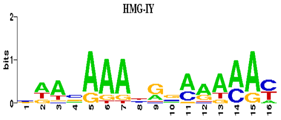
RREB1	1.8612e-02	--NKTGTGTNTGYGTRTGTG<br>NGKKKGKGGGTGKTTTGGGG	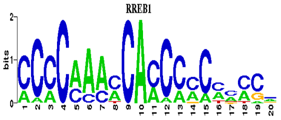
T	1.9860e-02	CACAYACRCANACACAMN<br>--TTCACACCTAG-----	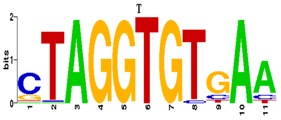
Foxd3	3.0420e-02	CACAYACRCANACACAMN<br>-AAANAAACAWTN-----	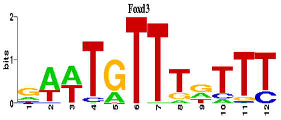
FOXI1	3.4243e-02	---NKTGTGTNTGYGTRTGTG<br>NNNTRTTTRTWT---------	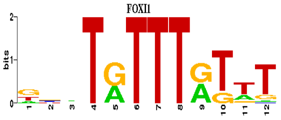
ZNF354C	5.0221e-02	NKTGTGTNTGYGTRTGTG<br>---GTGGAK---------	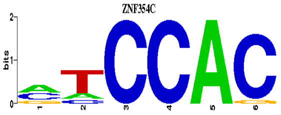
Foxq1	6.9039e-02	-NKTGTGTNTGYGTRTGTG<br>NATTGTTTATW--------	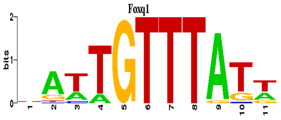
Pax6	6.9676e-02	NKTGTGTNTGYGTRTGTG<br>--MANTSAWGCGTGAA--	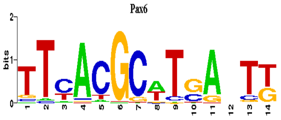

Motif_3

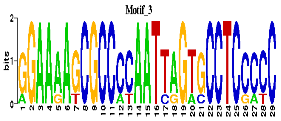

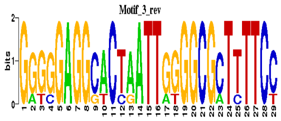

forward *reverse compliment*

Name	*E value*	*Alignment*	*Motif*
Pax4	4.8136e-11	-GGGGGAGGCACTAATTGGGGCGCTTTTCC<br>GKRNNNNKNNNNNNNNNNNKNNWWWTWTTY	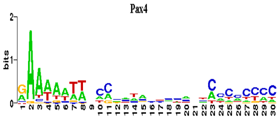
Pdx1	6.0028e-06	GGAAAAGCGCCCCAATTAGTGCCTCCCCC<br>-------------AATTAG----------	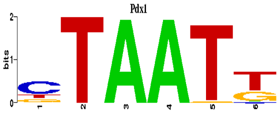
Prrx2	1.3008e-04	GGGGGAGGCACTAATTGGGGCGCTTTTCC<br>-----------TAATT-------------	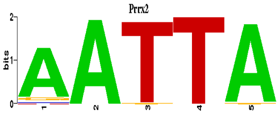
Dof3	1.8685e-04	GGGGGAGGCACTAATTGGGGCGCTTTTCC<br>--------------------NRCTTT---	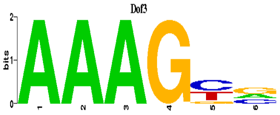
MNB1A	2.2225e-04	GGGGGAGGCACTAATTGGGGCGCTTTTCC<br>---------------------NCTTT---	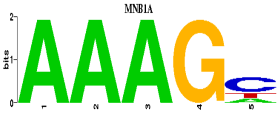
dl_1	3.1884e-04	GGGGGAGGCACTAATTGGGGCGCTTTTCC-<br>------------------SGGGTTTTTCCN	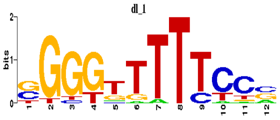
PBF	3.2872e-04	GGGGGAGGCACTAATTGGGGCGCTTTTCC<br>---------------------RCTTT---	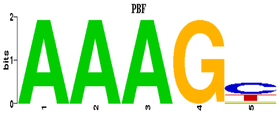
Agamous	3.7219e-04	GGAAAAGCGCCCCAATTAGTGCCTCCCCC<br>---------NCCNNWTTWGG---------	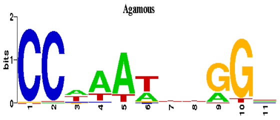
Nkx2-5	4.7794e-04	GGAAAAGCGCCCCAATTAGTGCCTCCCCC<br>------------CAMTTAW----------	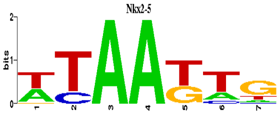
ATHB5	5.6424e-04	GGGGGAGGCACTAATTGGGGCGCTTTTCC<br>---------AATMATTGN-----------	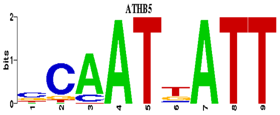

Motif_11

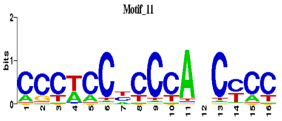

forward

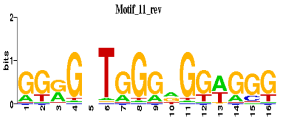

reverse compliment

Name	*E value*	*Alignment*	*Motif*
Pax4	6.5278e-04	GGRGNTGGGNGGWGGG--------------- -GKRNNNNKNNNNNNNNNNNKNNWWWTWTTY	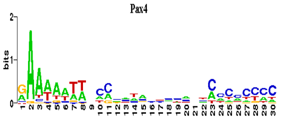
RREB1	1.3388e-03	-----GGRGNTGGGNGGWGGG NGKKKGKGGGTGKTTTGGGG-	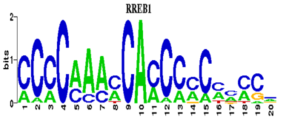
Klf4	9.3992e-03	CCCWCCNCCCANCYCC CCTTYYYTTN------	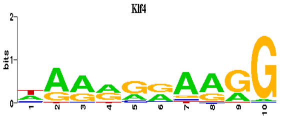
MZF1_1-4	2.0693e-02	CCCWCCNCCCANCYCC ---TCCCCN-------	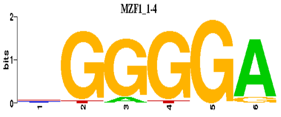
SP1	2.5923e-02	CCCWCCNCCCANCYCC ------ACCCNGCCNN	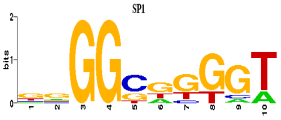
MZF1_5-13	3.3083e-02	CCCWCCNCCCANCYCC --NTNCCCCTMN----	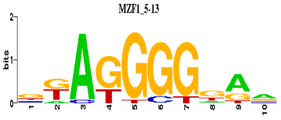
ZNF354C	6.7150e-02	CCCWCCNCCCANCYCC --MTCCAC--------	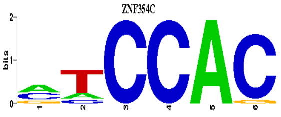
ZEB1	7.1363e-02	GGRGNTGGGNGGWGGG --------NAGGTG--	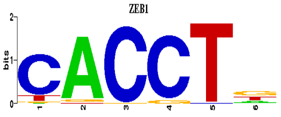
TFAP2A	7.6314e-02	GGRGNTGGGNGGWGGG ---GCCNNNNGS----	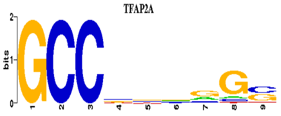
sna	8.2609e-02	GGRGNTGGGNGGWGGG --------CAGGTG--	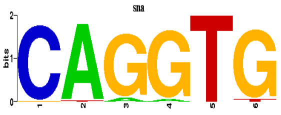

Motif_9

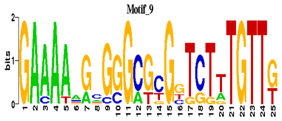 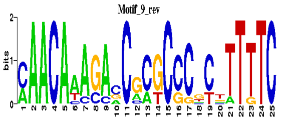

forward *reverse compliment*

Name	*E value*	*Alignment*	*Motif*
MNB1A	3.1854e-04	CAACAAAGANCGCGCCCNCNTTTTC<br>-----------------NCTTT---	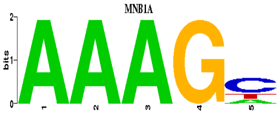
REST	6.5926e-04	----CAACAAAGANCGCGCCCNCNTTTTC<br>TCAGCACCWNGGACAGCNC----------	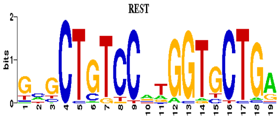
Dof3	8.5118e-04	CAACAAAGANCGCGCCCNCNTTTTC<br>----------------NRCTTT---	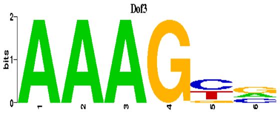
PBF	1.1530e-03	CAACAAAGANCGCGCCCNCNTTTTC<br>-----------------RCTTT---	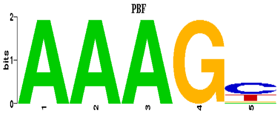
Dof2	2.6836e-03	CAACAAAGANCGCGCCCNCNTTTTC<br>----------------NGCTTT---	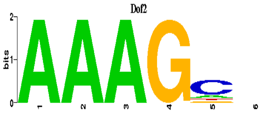
dl_1	7.0677e-03	CAACAAAGANCGCGCCCNCNTTTTC--<br>---------------SGGGTTTTTCCN	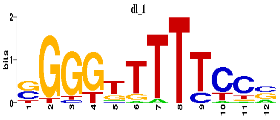
SP1	1.0486e-02	CAACAAAGANCGCGCCCNCNTTTTC<br>--------ACCCNGCCNN-------	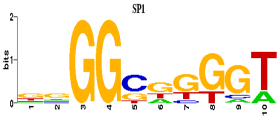
Sox5	1.3638e-02	GAAAANGNGGGCGCGNTCTTTGTTG<br>------------------ATTGTTN	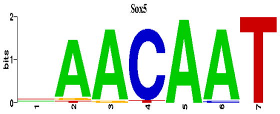
FOXI1	1.9751e-02	CAACAAAGANCGCGCCCNCNTTTTC<br>AWAYAAAYANNN-------------	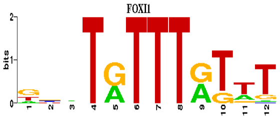
Foxd3	1.9823e-02	CAACAAAGANCGCGCCCNCNTTTTC<br>AAANAAACAWTN-------------	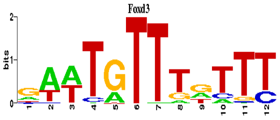

Motif_6

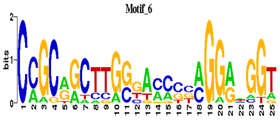

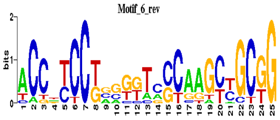

forward *reverse compliment*

Name	*E value*	*Alignment*	*Motif*
Eip74EF	3.0567e-04	ACCNYCCKNNKKTNSCAAGCYGCGG<br>--YTTCCGG----------------	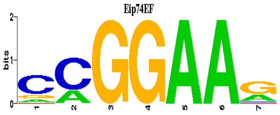
Roaz	4.4644e-04	ACCNYCCKNNKKTNSCAAGCYGCGG<br>GNMMCCYTRGGKKSC----------	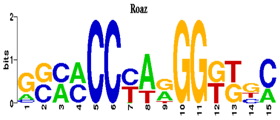
TEAD1	2.6430e-03	CCGCRGCTTGSNAMMNNNMGGRNGGT-<br>--------------CNSWGGAATGTR	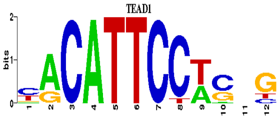
SPIB	1.2179e-02	ACCNYCCKNNKKTNSCAAGCYGCGG<br>---TTCCKST---------------	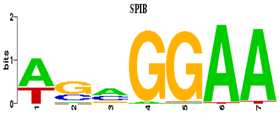
NHLH1	1.5149e-02	CCGCRGCTTGSNAMMNNNMGGRNGGT<br>NCGCAGCTGCGN--------------	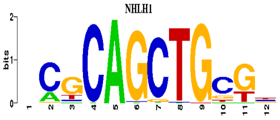
TFAP2A	1.6791e-02	ACCNYCCKNNKKTNSCAAGCYGCGG<br>----SCNNNNGGC------------	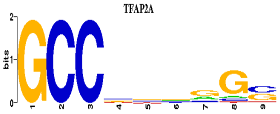
SPI1	1.7288e-02	ACCNYCCKNNKKTNSCAAGCYGCGG<br>--STTCCN-----------------	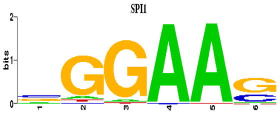
ELK4	1.9825e-02	ACCNYCCKNNKKTNSCAAGCYGCGG<br>-ACTTCCGGT---------------	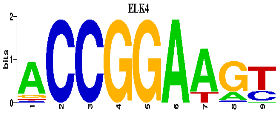
ELF5	3.1065e-02	CCGCRGCTTGSNAMMNNNMGGRNGGT<br>-----------------NMGGAARTN	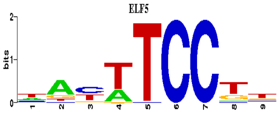
PBF	3.9398e-02	CCGCRGCTTGSNAMMNNNMGGRNGGT<br>-----RCTTT---------------	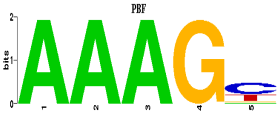

Motif_7

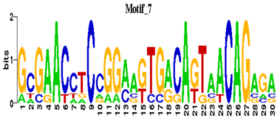

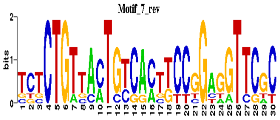

forward *reverse compliment*

Name	*E value*	*Alignment*	*Motif*
ELK4	2.2790e-06	TCTCTGTTACTGTCACTTCCGGAGGTTCGC<br>--------------ACTTCCGGT-------	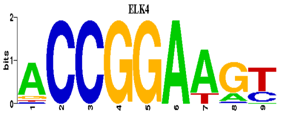
Eip74EF	3.0442e-06	TCTCTGTTACTGTCACTTCCGGAGGTTCGC<br>---------------YTTCCGG--------	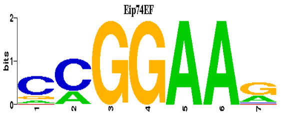
GABPA	4.9519e-06	GCGAACCTCCGGAAGTGACAGTAACAGAGA<br>-------ACCGGAAGNS-------------	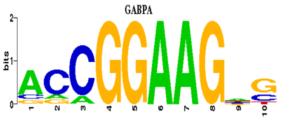
ovo	1.1286e-05	TCTCTGTTACTGTCACTTCCGGAGGTTCGC<br>--NCNGTTACW-------------------	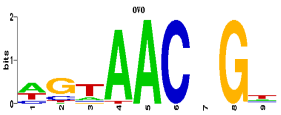
Spz1	2.2719e-04	TCTCTGTTACTGTCACTTCCGGAGGTTCGC<br>--GCTGWTACCCT-----------------	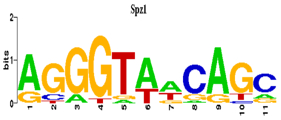
IRF2	4.8531e-04	TCTCTGTTACTGTCACTTCCGGAGGTTCGC<br>---NNWWNRSTTTCRCTTTCS---------	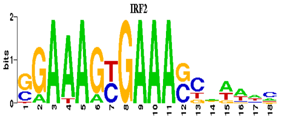
MIZF	9.0722e-03	TCTCTGTTACTGTCACTTCCGGAGGTTCGC<br>------------------GCGGACGTTN--	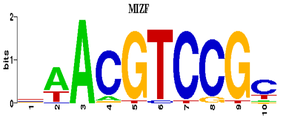
ELK1	9.6048e-03	TCTCTGTTACTGTCACTTCCGGAGGTTCGC<br>---------------YTTCCGGNNN-----	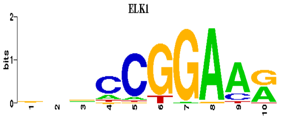
SPI1	1.8421e-02	TCTCTGTTACTGTCACTTCCGGAGGTTCGC<br>---------------STTCCN---------	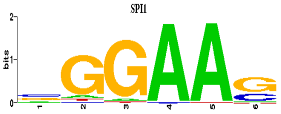
IRF1	1.8953e-02	TCTCTGTTACTGTCACTTCCGGAGGTTCGC<br>--------GGTTTCRNTTTN----------	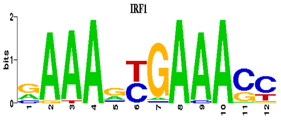

Sequence logo generation powered by weblogo
STAMP is written by Shaun Mahony
